# Functionalisation of conjugated macrocycles with type I and II concealed antiaromaticity *via* cross-coupling reactions†

**DOI:** 10.1039/d3me00045a

**Published:** 2023-05-10

**Authors:** Troy L. R. Bennett, Adam V. Marsh, James M. Turner, Felix Plasser, Martin Heeney, Florian Glöcklhofer

**Affiliations:** a Department of Chemistry and Centre for Processable Electronics, Imperial College London, Molecular Sciences Research Hub London UK f.glocklhofer@imperial.ac.uk; b KAUST Solar Center (KSC), Physical Science and Engineering Division (PSE), King Abdullah University of Science and Technology (KAUST) Thuwal Saudi Arabia; c Department of Chemistry, Loughborough University Loughborough LE11 3TU UK

## Abstract

Conjugated macrocycles can exhibit concealed antiaromaticity; that is, despite not being antiaromatic, under specific circumstances, they can display properties typically observed in antiaromatic molecules due to their formal macrocyclic 4*n* π-electron system. Paracyclophanetetraene (PCT) and its derivatives are prime examples of macrocycles exhibiting this behaviour. In redox reactions and upon photoexcitation, they have been shown to behave like antiaromatic molecules (requiring type I and II concealed antiaromaticity, respectively), with such phenomena showing potential for use in battery electrode materials and other electronic applications. However, further exploration of PCTs has been hindered by the lack of halogenated molecular building blocks that would permit their integration into larger conjugated molecules by cross-coupling reactions. Here, we present two dibrominated PCTs, obtained as a mixture of regioisomers from a three-step synthesis, and demonstrate their functionalisation *via* Suzuki cross-coupling reactions. Optical, electrochemical, and theoretical studies reveal that aryl substituents can subtly tune the properties and behaviour of PCT, showing that this is a viable strategy in further exploring this promising class of materials.

## Abstract 2

Halogenated paracyclophanetetraenes (PCTs) for the integration of the PCT motif into larger conjugated molecules by cross-coupling reactions are introduced.

## Graphical abstract

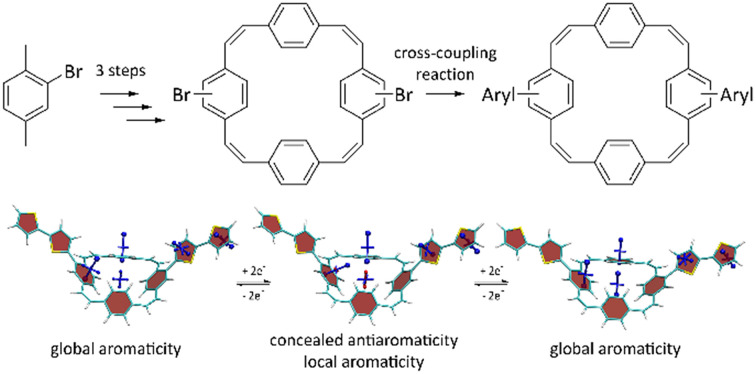

**Design, System, Application**

Paracyclophanetetraene (PCT) is a macrocycle derived from [24]annulene, which is an antiaromatic conjugated system with 4*n* π-electrons. However, the antiaromatic character of this system is concealed by the additional vinylene bridges, which introduce four locally aromatic units with 4*n* + 2 π-electrons that dominate the overall structure. This approach to concealing the antiaromaticity increases the stability of the molecule while preserving its potential to become globally aromatic upon twofold reduction or photoexcitation, leading to exceptional redox behaviour and unique photophysical properties. In this study, we employed PCT as the macrocyclic core and introduced two bromo groups to facilitate further functionalisation *via* cross-coupling reactions. We demonstrated the efficacy of this approach by performing Suzuki cross-coupling reactions with borylated thiophenes and bithiophene. The dibrominated PCTs are expected to serve as versatile molecular building blocks for the integration of the PCT motif into larger conjugated molecules, including the backbone of conjugated polymers. Such modifications can help to tailor important parameters like solubility and charge transport properties, making these molecules highly relevant to organic electronic materials applications.

## Introduction

**Scheme 1:**
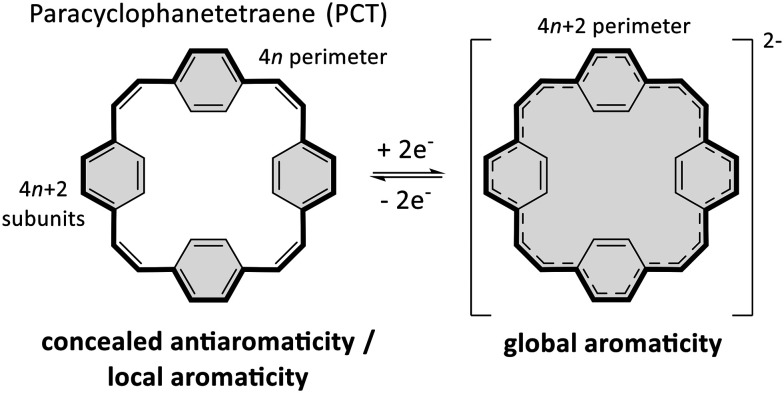
Redox reaction and aromaticity switching of paracyclophanetetraene (PCT; grey shadings indicate aromaticity). In the neutral state, the antiaromaticity of the formal macrocyclic 24 (4*n*) π-electron system (bold bonds) is concealed by the locally aromatic subunits, but the dianion can still become globally aromatic.

Conjugated macrocycles often feature interesting photophysical, electrochemical and magnetic properties as well as unusual intermolecular interactions, which can be attributed to their macrocyclic conjugated system and geometry.^1^ The conjugated system enables π-electron delocalisation and the emergence of global aromaticity under certain conditions. Paracyclophanetetraene (PCT, Scheme 1 left) and its derivatives are among the most interesting macrocycles in this regard,^2–7^ as they feature concealed antiaromaticity.^8^ Despite the formal macrocyclic system of 24 (4*n*) π-electrons along the perimeter of the molecule (Scheme 1 left, bold bonds), PCT and its derivatives are not antiaromatic but dominated by the local aromaticity of the four phenylene subunits with 6 (4*n* + 2) π-electrons, which obey Hückel's rule and conceal the antiaromaticity of the macrocyclic conjugated system. Nevertheless, the macrocycles can still exhibit behaviour under certain conditions that would normally be expected for antiaromatic molecules.

Most interestingly, PCT and its derivatives can become globally aromatic in redox reactions, as twofold oxidation or reduction results in a macrocyclic conjugated system of either 22 or 26 (4*n* + 2) π-electrons and enhanced macrocyclic π-electron delocalisation (Scheme 1). Such behaviour, defined as type I concealed antiaromaticity, was shown to be particularly interesting for use as battery electrode materials, as switching between different aromatic states can enable excellent redox properties.^4^

PCT and its derivatives are also considered to feature type II concealed antiaromaticity,^8^ meaning that the macrocycles can become globally aromatic in the lowest singlet and/or triplet excited states, where the electron counting rules are reversed according to what is known as Baird's rule.^9–11^ In these states, molecules with 4*n* π-electrons in a conjugated system can be expected to be aromatic and with 4*n* + 2 π-electrons to be antiaromatic. Molecules exhibiting type II concealed antiaromaticity are interesting for various applications, ranging from singlet fission to photoresponsive materials and molecular probes.^12–14^

In addition to type I and II concealed antiaromaticity, the PCT (sub)structure has been shown to lead to high degrees of porosity in the solid state, a result of its macrocyclic geometry.^4,7^ The porosity is believed to facilitate the insertion of counterions when used as battery electrode materials and to prevent unwanted volume expansion upon insertion.

**Scheme 2:**
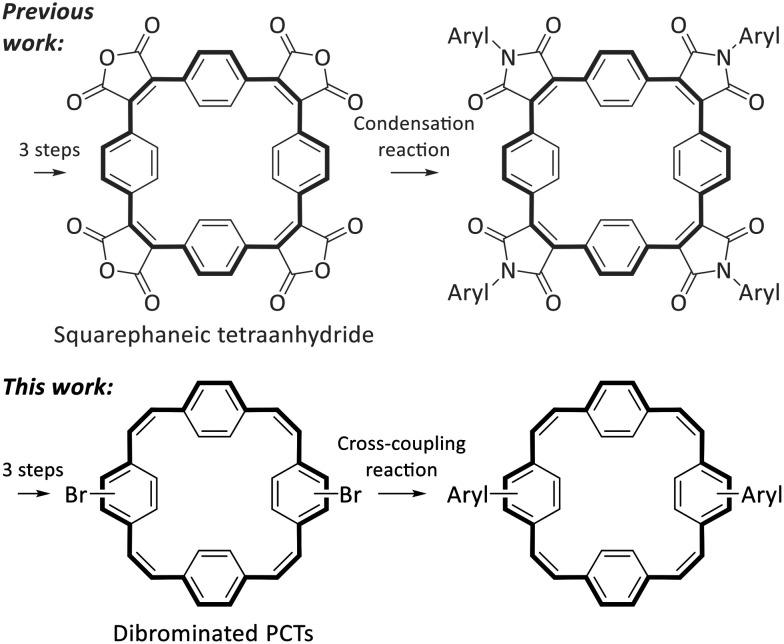
Previous work: squarephaneic tetraanhydride as a building block for the synthesis of molecules with cross-conjugated imide groups between the PCT core and the aryl groups. This work: dibrominated PCTs as building blocks for the synthesis of molecules with linear conjugation between the PCT core and the aryl groups.

Despite these interesting properties of PCT and its derivatives, suitable reactive building blocks for the use of PCT as a structural motif in more complex organic systems were missing from the literature. In a first step to fill this gap, we recently introduced squarephaneic tetraanhydride (Scheme 2 top left), which features PCT as a substructure as well as four reactive carboxylic anhydride groups for further chemical functionalisation.^7^ Besides other reactions, the anhydride groups can be converted efficiently into imide groups by condensation reactions with arylamines (Scheme 2 top), allowing the synthesis of a wide range of compounds with targeted functionality. However, in the product of this reaction, the aryl groups and the PCT core are linked *via* cross-conjugated imide groups. While the cross-conjugated nature of this linking group does not matter for some applications (or may even be beneficial), it does affect some of the molecular properties. Most notably, cross-conjugation reduces the electronic communication and charge transport between the linked parts of the molecules (compared to linear conjugation).^15,16^ For some applications, especially in organic electronics, linear conjugation is, therefore, an important requirement for good performance, *e.g.*, good charge carrier mobility. Thus, we were interested in developing a reactive building block with a PCT substructure that allows for the synthesis of molecules with linear conjugation between aryl groups and the PCT core.

In the synthesis of organic electronic materials, linear conjugation between different parts of a molecule is most commonly achieved by coupling reactions, often Pd-catalysed Suzuki cross-coupling reactions. These reactions usually require halogenated building blocks, also for the synthesis of the borylated precursors. Therefore, for the present work, we aimed to develop halogenated PCTs that can be used for such cross-coupling reactions (Scheme 2 bottom). Specifically, we aimed to (i) identify a straightforward route to dibrominated PCTs; (ii) demonstrate their use in Suzuki cross-coupling reactions with thiophene and bithiophene precursors (yielding molecules with linear conjugation between the aryl groups and the PCT core); and (iii) investigate the properties of the cross-coupling products, including how the linear conjugation between the additional, locally aromatic thienyl/bithienyl units and the PCT core affects the redox properties and the ability of the macrocyclic core to become globally aromatic in the doubly charged and excited states.

## Results and discussion

### Synthesis

**Scheme 3:**
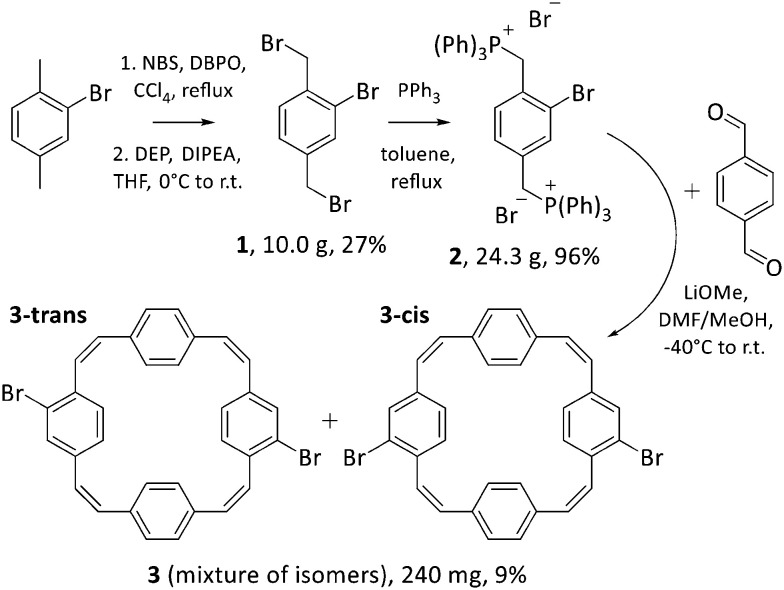
Synthesis of dibrominated PCTs 3**-*cis*** and 3**-*trans***, which were isolated as mixture 3. Reactants: *N*-bromosuccinimide (NBS), dibenzoyl peroxide (DBPO), diethylphosphite (DEP), *N*,*N*-diisopropylethylamine (DIPEA), triphenylphosphine (PPh_3_), lithium methoxide (LiOMe).

Squarephaneic tetraanhydride, the previously developed building block with a PCT substructure, was obtained in good yield by a Perkin-type cyclisation reaction.^7^ However, this method is not suitable for the synthesis of halogenated PCTs without further substituents, as it inevitably yields molecules with anhydride groups. Hence, we opted for a Wittig cyclisation reaction for the synthesis of our halogenated PCTs. The method has been used previously for the synthesis of unsubstituted PCT,^4,17^ tetrabrominated PCT with two *para*-dibrominated phenylene units,^18^ various iodinated PCTs,^19,20^ as well as methoxy- and methylthio-substituted PCTs.^6^ Iodinated PCTs were shown to undergo intramolecular reactions, whereas the tetrabrominated PCT can be expected to deviate strongly from planarity after coupling reactions (for steric reasons), which is detrimental for the occurrence of global aromaticity in the charged or excited states. To avoid these issues, we decided to target novel dibrominated PCTs 3**-*cis*** and 3**-*trans*** (Scheme 3 bottom), but *via* the same synthetic route as used for the synthesis of other halogenated PCTs.

In the first step of the three-step synthetic route towards 3**-*cis*** and 3**-*trans*** (Scheme 3), 2-bromo-1,4-dimethylbenzene was brominated in the benzylic positions with *N*-bromosuccinimide (NBS) in the presence of the radical initiator dibenzoyl peroxide (DBPO) following a published protocol.^21^ Contrary to the previous report, the dibrominated compound 1 formed in this reaction proved difficult to isolate due to the presence of over-brominated side products that could not be separated by silica gel chromatography. This was resolved by treating the crude mixture with diethylphosphite (DEP) and *N*,*N*-diisopropylethylamine (DIPEA) in THF, which can convert these side products to compound 1.^22^ Following this treatment, compound 1 was isolated in 27% yield. The bromomethyl groups were then converted into phosphonium groups through a straightforward substitution reaction with PPh_3_, affording Wittig reagent 2 in almost quantitative yields.

**Scheme 4:**
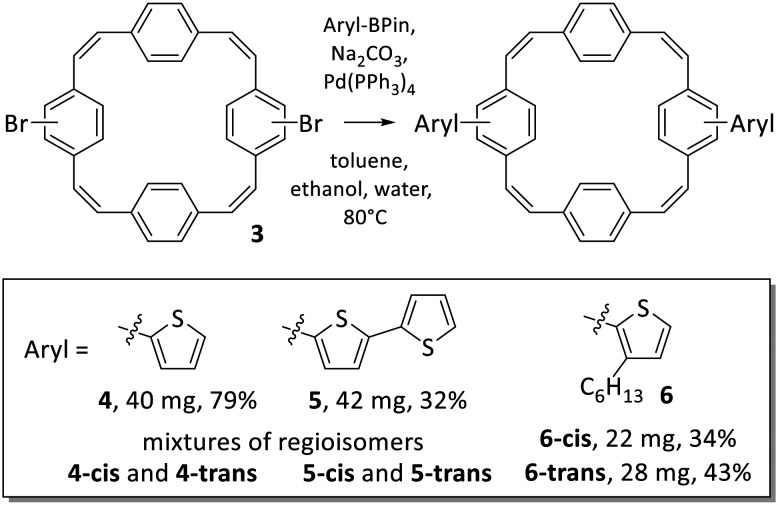
Suzuki cross-coupling reactions of the mixture of dibrominated PCT isomers 3 and the respective boronic acid pinacol esters (Aryl-BPin), yielding dithienyl-substituted PCT 4 and bis(bithienyl)-substituted PCT 5 as mixtures of isomers and bis(3-hexylthienyl)-substituted PCT 6 as separated isomers 6**-*cis*** and 6**-*trans*** following purification by recycling preparative GPC.

For the formation of dibrominated PCTs 3**-*cis*** and 3**-*trans***, the Wittig reagent 2 was reacted with terephthaldehyde and, as expected, this cyclisation reaction produced a mixture of the two dibrominated PCT regioisomers. Attempts to separate the isomers by column chromatography, recrystallisation, or recycling preparative gel permeation chromatography (GPC) were, however, unsuccessful. The two isomers were obtained as mixture 3 upon purification by recycling preparative GPC (in yields similar to those of other halogenated PCTs) and used in this form for further modification by Suzuki cross-coupling reactions (Scheme 4).

**Fig. 1:**
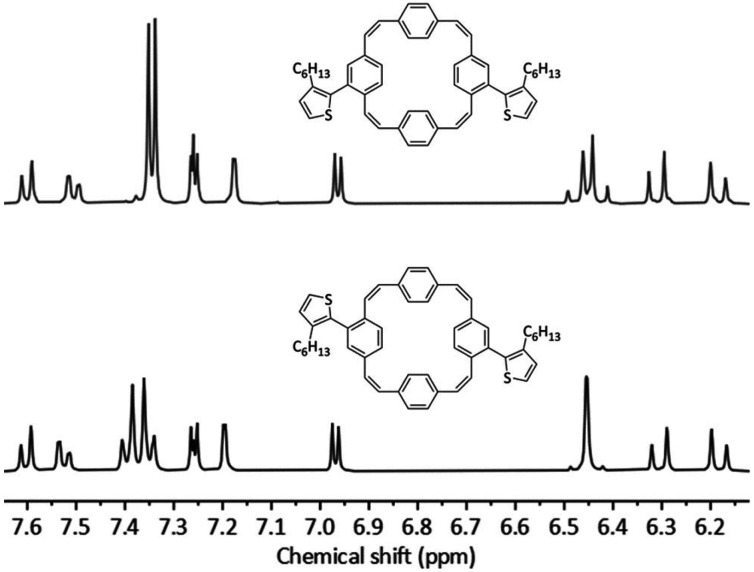
^1^H NMR (CDCl_3_, 400 MHz) spectra and molecular structures of 6**-*cis*** (top) and 6**-*trans*** (bottom).

The initial coupling reactions were performed using isomer mixture 3 and thiophene-2-boronic acid pinacol ester as the coupling partners for the synthesis of compound 4. As for compound 3, recycling preparative GPC efficiently removed all impurities of the reaction but was unable to separate the two isomers, affording dithienyl-substituted PCT 4 as a mixture of isomers in a yield of 79%. Curious as to whether an increased size of the substituents may enable separation of the isomers by recycling GPC, we then synthesised bis(bithienyl)-substituted PCT 5 and bis(3-hexylthienyl)-substituted PCT 6, again using mixture 3 and the respective boronic acid pinacol ester as coupling partners. While separation of the two isomers of compound 5 could not be achieved, separation proved feasible for compound 6, enabling individual characterisation of the two isomers 6**-*cis*** and 6**-*trans***. The two isomers could be easily distinguished and assigned based on their ^1^H NMR spectra (Fig. 1), which showed the phenylene-Hs of the two unsubstituted phenylene units as two singlets in the case of 6**-*cis*** (at 7.34 and 7.35 ppm, 4H each) and as two doublets in the case of 6**-*trans*** (at 7.40 and 7.35 ppm, 4H each). These differences in the ^1^H NMR spectra are a consequence of the differences in symmetry of the two compounds.

The identities of all new compounds were confirmed by ^1^H and ^13^C{^1^H} NMR spectroscopy as well as high-resolution mass spectrometry (HRMS), except for compound 5 which was characterised by NMR spectroscopy and MALDI-MS. Thermogravimetric analysis (TGA) of compounds 3 and 6**-*cis*** (as a representative of a coupling product) further revealed decomposition temperatures of 227 °C and 354 °C, respectively.

### UV-vis absorption and photoluminescence

**Fig. 2:**
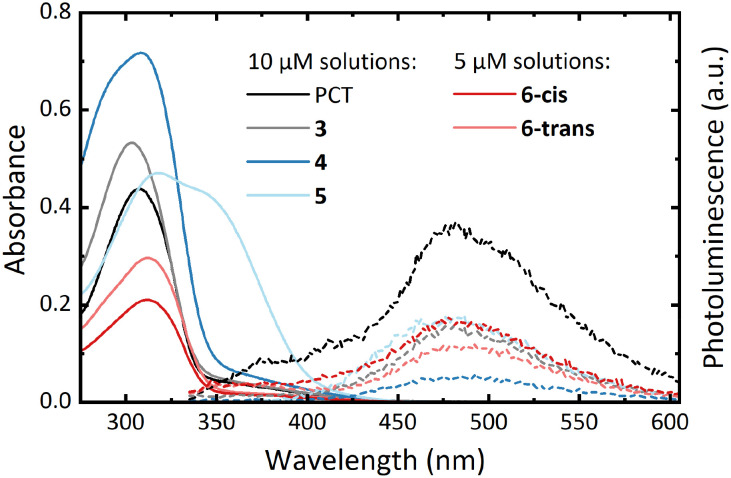
UV-vis absorption (solid lines) and photoluminescence spectra (dashed lines) of the macrocycles in CHCl_3_ solution (approx. 5 and 10 μM). For recording the PL spectra, the macrocycles were excited at their absorption maximum wavelengths.

UV-vis absorption measurements in CHCl_3_ solution showed similar spectra for all compounds except for the bithienyl-substituted compound 5 (Fig. 2, solid lines). Compared to PCT (*λ*_abs_ = 307 nm), the spectra showed a minor blueshift of the absorption maximum for compound 3 (*λ*_abs_ = 303 nm) and a minor redshift for compounds 4 (*λ*_abs_ = 308 nm), 6**-*cis*** (*λ*_abs_ = 311 nm) and 6**-*trans*** (*λ*_abs_ = 312 nm). For compound 5 (*λ*_abs_ = 319 nm), a more significant redshift was observed as well as another absorption band at approx. 340 nm.

In addition to these solution measurements, thin-films of the compounds were prepared *via* spin-coating. The measurements of these films revealed only a small redshift of the absorption maxima compared to the solution measurements (see ESI,† Table S1 and Fig. S24).

All macrocycles, including PCT, exhibit photoluminescence (PL) maxima at around 475 nm (Fig. 2, dashed lines). The similarity of the emission spectra of PCT and the substituted macrocycles suggests that the excitation is concentrated on the PCT core in the substituted macrocycles, with only a weak effect of the aryl substituents. However, the macrocycles are very weak emitters, with a determination of the photoluminescence quantum yield of PCT previously indicating a value of <0.1%.^6^ In contrast to this previous work, where the introduction of methoxy and methylthio substituents at the PCT core was shown to increase the PL intensity, the introduction of aryl substituents did not result in such an increase. Nonetheless, as for PCT and the methoxy- and methylthio-substituted PCTs, the energy difference between the absorption and emission maximum was found to be large for all macrocycles (between 1.50 and 1.25 eV), a signature of excited-state aromaticity and type II concealed antiaromaticity (*cf.* ref. 5 and 23). As in the absorption spectra, the two separated isomers, 6**-*cis*** and 6**-*trans***, exhibited no considerable differences in the emission spectra.

### Electrochemistry

**Fig. 3:**
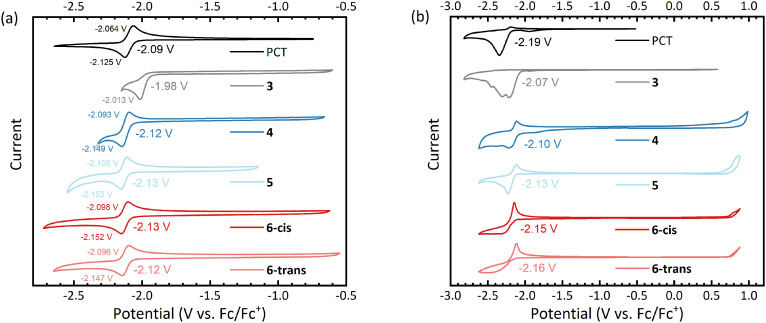
Cyclic voltammograms of the macrocycles (a) in dimethylformamide (DMF) solution, and (b) in thin films dropcast onto the working electrode. The voltammograms were recorded using a glassy carbon working electrode, a platinum counter electrode, and a silver wire quasi-reference electrode (QRE) at a scan rate of 0.1 V s^−1^. 0.1 M [*n*-Bu_4_N]PF_6_ solutions in either DMF (solution) or acetonitrile (thin film) were used as the supporting electrolyte.

Cyclic voltammetry (CV) measurements of the macrocycles in dimethylformamide solution showed reversible reduction processes at slightly lower (more negative) redox potentials than for PCT (Fig. 3a), except for the dibrominated intermediate 3, which underwent irreversible reduction at a higher (less negative) potential. For all aryl-substituted PCTs, the difference between the cathodic and anodic peak potential was below the thermodynamic limit for a one-electron process (57 mV at 25 °C), indicating that the observed redox reactions are two-electron processes. This corresponds well with type I concealed antiaromaticity and the expected transition to a globally aromatic state upon twofold reduction.

To investigate the redox behaviour in the solid state, we measured thin films dropcast onto the working electrode and then immersed in an acetonitrile-based electrolyte (Fig. 3b). Similar to the solution measurements, the thin film measurements showed reversible reduction for all aryl-substituted PCTs. In contrast, the reduction of the unsubstituted PCT thin film was irreversible, which can be explained by the visually observed dissolution of the reduced PCT species in the electrolyte. The thin film measurements gave redox potentials similar to those in solution, but the values in Fig. 3b should be taken with caution, as the ferrocene/ferrocene^+^ (Fc/Fc^+^) redox potential for referencing had to be obtained from a separate measurement after cleaning the working electrode (see ESI† for further details).

While the reduction was found to be reversible for the aryl-substituted PCTs in this work, only irreversible oxidation was observed. The same observation was made previously for methoxy-/methylthio-substituted PCTs.^6^

### Computations

To study how the linear conjugation between the additional, locally aromatic thienyl/bithienyl units and the PCT core affects the local and global aromaticity in the neutral, doubly charged, and excited states of the molecules, we carried out nucleus-independent chemical shift (NICS)^24^ calculations for the different states. The NICS tensors were then represented graphically using the visualisation of chemical shielding tensors (VIST) method.^25^ VIST allows the visualisation of the chemical shielding in the context of the molecular structure by showing the shielding tensor components as blue (shielded, aromatic) or red (deshielded, antiaromatic) dumbbells, each relating to ring currents in a plane perpendicular to it. The method does not require the *a priori* definition of an external magnetic field and can, therefore, provide an unbiased view of currents in different planes, a highly valuable feature when studying non-planar multiring systems, such as the molecules in this work.

In addition to the VIST plots, we analysed the electronic structure in more detail by computing the natural difference orbitals (NDOs)^26^ between the neutral S_0_ state and the doubly charged (2+ and 2−) and excited triplet states (T_1_) as well as the natural transition orbitals (NTOs)^27^ to the excited singlet state (S_1_). The NDOs/NTOs can show whether the charge or the excitation is delocalised over the entire molecule, predominantly delocalised over the PCT core, or localised on a certain part of the molecule. Importantly, while VIST provides information regarding the magnetic criterion of aromaticity, NDOs/NTOs can provide information regarding the electronic criterion.^28^

**Fig. 4:**
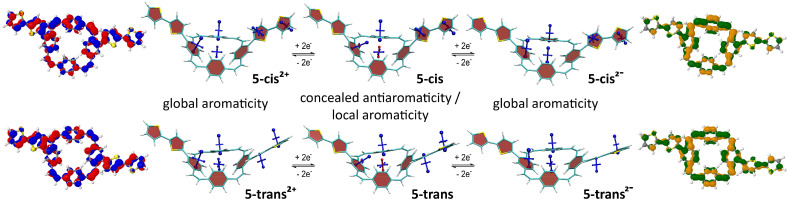
Analysis of the neutral and doubly charged states for 5**-*cis*** and 5**-*trans***. Centre: VIST plots. Left and right: Dominant natural difference orbitals (NDOs) between the neutral states and the dications (blue/red NDOs for electron detachment) and the dianions (green/orange NDOs for attachment). See ESI† Fig. S25 for the analogous plots of 4**-*cis*** and 4**-*trans***.

Fig. 4 shows the VIST plots for 5**-*cis*** and 5**-*trans*** in the neutral and doubly charged states as well as the detachment NDOs (blue/red) for the 2+ states and attachment NDOs (green/orange) for the 2− states. The VIST plots of the neutral molecules show the local aromaticity of the phenylene subunits of the PCT core and the thienylene and thienyl units of the substituents by large blue tensor components that are almost perpendicular to the planes of these units. For both molecules, the tensor at the centre of the PCT core shows only a small deshielded component (NICS of about +11 ppm). Previous analysis^5,25^ suggests that such deshielding derives from the phenylene rings and does not actually reflect global antiaromaticity, indicating that the antiaromaticity of the macrocyclic system is well concealed. In the doubly charged states, however, the main component of the tensors at the phenylene units are tilted and almost perpendicular to the plane of the PCT core, showing that the local aromaticity of the subunits is strongly perturbed by macrocyclic diatropic (aromatic) currents. In contrast, the shielding tensors at the substituents do not show any drastic changes, but they indicate slightly reduced shielding, which can be explained by deshielding effects on the outside of the macrocyclic diatropic currents (see ESI† Fig. S26 for further details). On the inside, the macrocyclic currents result in a shielded, aromatic tensor component perpendicular to the plane of the PCT core. Interestingly, this component was found to be considerably larger in the dianions (−20.6 ppm in 5**-*cis*** and −22.0 ppm in 5**-*trans***) than in the dications (−9.0 ppm and −10.8 ppm, respectively), suggesting that the global aromaticity is stronger in the dianions. The charged-state aromaticity is also reflected in the shape of the NDOs. The formal HOMO (left; blue/red) and LUMO (right; green/orange) are both fully delocalized around the PCT core and possess 12 nodal planes around the perimeter, forming a pair of degenerate orbitals in the limit of perfect cyclical symmetry. Aromaticity and diatropic ring currents are now associated with either occupying none (as in the dication) or both (as in the dianion) of these quasi-degenerate orbitals.^28,29^ Moreover, the NDOs provide an explanation for the differences in global aromaticity by showing that the positive charge of the dications is delocalised over the entire molecule, whereas the negative charge of the dianions is concentrated on the PCT core. Therefore, the dianions behave more like isolated PCT, with the additional two electrons resulting in the formation of a macrocyclic 4*n* + 2 π-electron system.

The computations of 4**-*cis*** and 4**-*trans*** and their doubly charged states (ESI† Fig. S25) revealed very similar effects, but the NDOs show that in both the dianions and the dications, the charge is predominantly concentrated on the PCT core, with less delocalisation to the substituents than in 5**-*cis*** and 5**-*trans***. Consequently, the differences in global aromaticity between the dianions and dications are less pronounced.

**Fig. 5:**
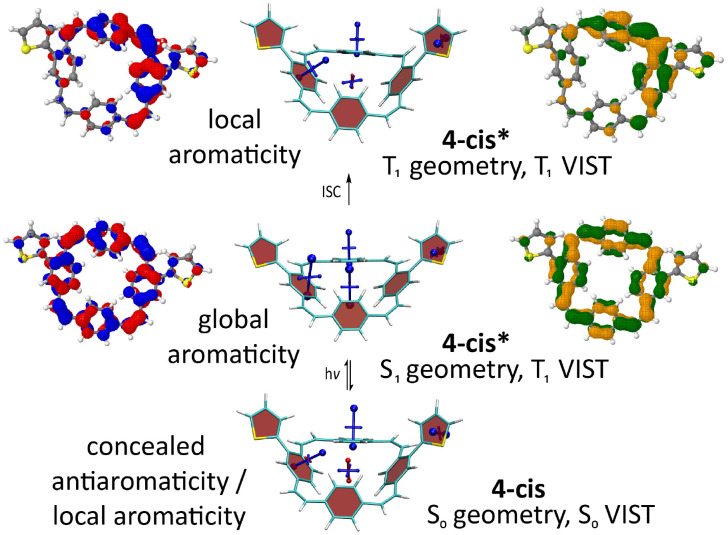
Analysis of the S_0_, S_1_, and T_1_ states for 4**-*cis***. Centre: VIST plots (using T_1_ also at the S_1_ geometry). Left and right: Dominant natural transition/difference orbitals (NTOs/NDOs) for the S_1_ and T_1_ states (blue/red for electron detachment; green/orange for attachment). See ESI† Fig. S27–S29 for the analogous plots of 4**-*trans***, 5**-*cis***, and 5**-*trans***.

Computations of the S_1_ state provided a similar picture for all molecules. As shown for 4**-*cis*** (Fig. 5) and the other molecules (ESI† Fig. S27–S29), the detachment and attachment NTOs indicate that the hole and the electron of the exciton are predominantly delocalised over the PCT core in the S_1_ state, which explains the similarity of the fluorescence spectra of the molecules. The perfect cyclic delocalisation of the NTOs along with their nodal structure (12 nodal planes for both HOMO and LUMO) serves as an indicator of excited-state aromaticity.^28^ As the computation of shielding tensors in the S_1_ state is not routinely possible, we computed VIST plots of the T_1_ states at the S_1_ geometry instead. For all four molecules, these plots indicate pronounced macrocyclic diatropic currents. As in the doubly charged states, the local aromaticity of the thienyl and bithienyl substituents is largely retained.

In the T_1_ state, the computations for 4**-*cis*** indicate a localised state (Fig. 5 top). According to the NDOs, the symmetry is broken, and the excitation is localised on one of the vinylene groups, which adopts a twisted structure. The VIST plots indicate the local aromaticity of the phenylene subunits and the substituents, with no considerable contributions from macrocyclic ring currents. Similar effects were previously described for methoxy- and methylthio-substituted PCTs.^6^ For 5**-*cis*** and 5**-*trans***, the computations show similar but slightly less pronounced symmetry breaking and loss of global aromaticity in the T_1_ state (ESI† Fig. S28 and 29). In contrast, the computations suggest that 4**-*trans*** retains its global aromaticity in the T_1_ state (ESI† Fig. S27), with the excitation delocalised over the PCT core according to the NDOs. To understand this difference, it is worth noting that the molecules studied may well possess several local T_1_ minima with different degrees of aromaticity. However, a full characterisation of the conformational space and all local minima is beyond the scope of this work.

## Conclusions

Our results show that dibrominated PCTs for functionalisation *via* cross-coupling reactions can be obtained *via* a straightforward three-step synthetic route, with a Wittig cyclisation reaction as the key step. Although the two dibrominated PCT regioisomers obtained from the cyclisation reaction could not be separated, they can be used as a mixture in Suzuki cross-coupling reactions, as demonstrated by coupling reactions with different borylated thiophene and bithiophene precursors. In the case of the coupling reaction with 3-hexylthiophene-2-boronic acid pinacol ester, the two isomers 6**-*cis*** and 6**-*trans*** obtained from the coupling reaction could be separated by recycling preparative gel permeation chromatography (GPC) and characterised individually.

From the experimental characterisation of the coupling products, we can conclude that: (i) the functionalisation of PCT with thienyl or bithienyl groups only leads to minor changes in the PL. The effect on the UV-vis absorption was found to depend on the nature of the introduced group, with the introduction of bithienyl groups leading to a more intense absorption at longer wavelengths. (ii) The introduction of thienyl or bithienyl groups does not affect the reversibility and two-electron nature of the electrochemical reduction. As for PCT, a highly reversible two-electron reduction was found for the substituted PCT derivatives in the cyclic voltammetry (CV) measurements in solution. In the thin-film measurements, the aryl groups improved the reversibility of the reduction by decreasing the solubility of the reduced species in the electrolyte. (iii) The two separated isomers exhibit almost identical properties. Depending on the intended application of the molecules, a separation of the isomers may therefore not be necessary.

According to the computational characterisation, thienyl- and bithienyl-substituted PCTs are locally aromatic in the neutral ground state, but the PCT core becomes globally aromatic in the doubly charged states as well as in the lowest singlet excited state. Despite some differences in the strength of the macrocyclic ring currents depending on the substituents, the molecules can therefore be considered to feature both type I and II concealed antiaromaticity, a conclusion supported by the experimental findings. The situation is more complicated in the lowest triplet excited state, as symmetry breaking and a loss of global aromaticity can occur.

The dibrominated PCTs in our study are highly valuable building blocks for synthesizing other molecules with linear conjugation between PCT and the coupling partner. The building blocks permit the integration of the PCT motif into larger conjugated molecules, as demonstrated in this study, and potentially enable the synthesis of conjugated polymers containing macrocycles embedded in their backbone. This allows for a retention of the excellent redox properties that result from the concealed antiaromaticity of the PCT motif whilst customizing the solubility for the intended application and improving the charge transport properties that benefit from linear conjugation between different parts of a molecule, with relevance to a range of electronic applications.

## Conflicts of interest

There are no conflicts to declare.

## Supplementary Material

ME-008-D3ME00045A-s001
