# Supplementary material for: Functionalisation of conjugated macrocycles with type I and II concealed antiaromaticity via cross-coupling reactions
Source: Mol Syst Des Eng. 2023 May 10;8(6):713–20. doi: 10.1039/d3me00045a (PMC10243434; doi:10.1039/d3me00045a)
Supplement: ME-008-D3ME00045A-s001 [file ME-008-D3ME00045A-s001.pdf]

Electronic Supplementary Material (ESI) for Molecular Systems Design & Engineering.
This journal is © The Royal Society of Chemistry 2023

# Electronic Supplementary Information (ESI)

## Functionalisation of conjugated macrocycles with Type I and II concealed antiaromaticity via cross-coupling reactions

Troy L. R. Bennett,[a] Adam V. Marsh,[b] James M. Turner,[c] Felix Plasser,[c] Martin Heeney[b] and Florian Glöcklhofer[*a]

[a] Department of Chemistry and Centre for Processable Electronics, Imperial College London, Molecular Sciences Research Hub, London, UK.
Email: f.glocklhofer@imperial.ac.uk

[b] KAUST Solar Center (KSC), Physical Science and Engineering Division (PSE), King Abdullah University of Science and Technology (KAUST), Thuwal, Saudi Arabia

[c] Department of Chemistry, Loughborough University, Loughborough, LE11 3TU, UK

### Table of Contents

1. Synthesis ........ 2
2. $^{1}H$ and $^{13}C\{^{1}H\}$ NMR spectra ........ 9
3. Mass spectra ........ 17
4. Thermogravimetric analysis (TGA) ........ 20
5. Electrochemical measurements ........ 21
6. UV-vis absorption and photoluminescence measurements ........ 22
7. Computational analysis ........ 23
8. References ........ 28

# 1. Synthesis

All reagents were obtained from commercial sources and used as received. With the exception of carbon tetrachloride and methanol, all reaction solvents were obtained from a Grubbs-type solvent purification system and were sparged with nitrogen prior to use. All reactions were performed under nitrogen atmosphere using standard Schlenk line techniques, and work-ups were performed under air. Purification by recycling preparative gel permeation chromatography (GPC) was carried out on a LaboACE LC-5060 (Japan Analytical Industry Co., Tokyo, JAPAN) recycling GPC system equipped with a JAIGEL-2HR column and a TOYDAD800-S detector. $CH_2Cl_2$ was used as the eluent for the preparative recycling GPC in all cases, with a flow rate of 10 mL $min^{-1}$.

NMR spectra were recorded at room temperature on a Bruker Avance 400 MHz spectrometer and referenced to the residual solvent peaks of $CDCl_3$ or $CD_2Cl_2$ at 7.26 or 5.32 ($^1H$ NMR) and 77.16 or 53.84 ppm ($^{13}C\{^1H\}$ NMR) respectively. The NMR signals were fully assigned for the separated regioisomers **6-cis** and **6-trans** (where the assignment was possible) using 2D correlation spectroscopy. Coupling constants are measured in Hz.

## 1.1. 2-Bromo-1,4-bis(bromomethyl)benzene (1)

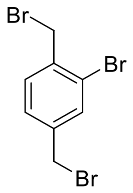


Molecular Weight: 342.86

Prepared using a previously reported method for the conversion of over-brominated side product.[1]

2-Bromo-1,4-dimethylbenzene (20.0 g, 108 mmol, 1.0 equiv.) was dissolved in carbon tetrachloride (100 mL) and degassed with $N_2$ for 1 hour. A separate flask was then charged with *N*-bromosuccinimide (NBS; 42.3 g, 238 mmol, 2.2 equiv.) and dibenzoyl peroxide (DBPO; 0.2 g, 0.864 mmol, 0.008 equiv.). The solution of 2-bromo-1,4-dimethylbenzene was added via cannula and the suspension was heated to reflux and stirred for 2 hours. The suspension was then allowed to cool to room temperature, filtered, and the solvent was removed *in vacuo*. To remove over-brominated species, the crude product was dissolved in THF (300 mL) and diethylphosphite (DEP; 32.8 g, 238 mmol, 2.2 equiv.) and *N*,*N*-diisopropylethylamine (DIPEA; 33.5 g, 259 mmol, 2.4 equiv.) were added. The solution was stirred vigorously at room temperature, and the reaction was monitored by taking an aliquot, removing the solvent *in vacuo*, and collecting a $^1H$ NMR spectrum, to check for consumption of the over-brominated species (which present indicative singlets at ~6.55 ppm). After 2 days, the reaction was filtered to

remove the yellow precipitate. The solvent of the solution was then removed *in vacuo*, and the residue was purified by silica gel column chromatography, eluting with hexane. The resulting near-pure product was recrystallized from the minimum amount of boiling hexane to give product **1** as white crystals (10.0 g, 29.2 mmol, 27%).

*Note: The step involving DEP and DIPEA produces a large amount of an insoluble yellow material. 300 mL of THF (or more) and a large stirrer bar are required to ensure vigorous stirring over the entire reaction time.*

Characterisation supported by previously reported synthesis of this molecule.[2] $^1$H NMR (400 MHz, $CDCl_3$): δ = 7.62 (d, *J* = 1.6 Hz, 1H), 7.43 (d, *J* = 8.0 Hz, 1H), 7.32 (dd, *J* = 7.6, 1.6 Hz, 1H), 4.58 (s, 2H), 4.41 (s, 2H) ppm.

## 1.2. 2-Bromo-1,4-bis(triphenylphosphonium)benzene dibromide (2)

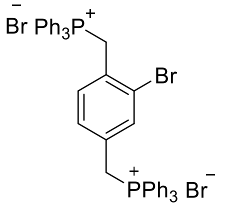


Molecular Weight: 867.44

Compound **1** (10.0 g, 29.2 mmol, 1.0 equiv.) and triphenylphosphine (30.6 g, 117 mmol, 4.0 equiv.) were dissolved in toluene (350 mL), heated to reflux, and stirred overnight, over which time a white precipitate formed. The solid was collected by filtering through a sintered funnel, and the solid was washed with toluene (3 x 200 mL) and dried *in vacuo* to yield product **2** as a fluffy white solid (24.3 g, 28.0 mmol, 96%).

Characterisation supported by previously reported synthesis of this molecule.[2] $^1$H NMR (400 MHz, $CDCl_3$): δ = 7.83-7.62 (m, 30H), 7.30 (m, 1H) 6.88 (m, 2H), 5.67 (d, *J* = 14.4 Hz, 2H), 5.44 (d, *J* = 14.4 Hz, 2H) ppm.

### 1.3. Dibrominated PCTs 3 (mixture of regioisomers 3-cis and 3-trans)

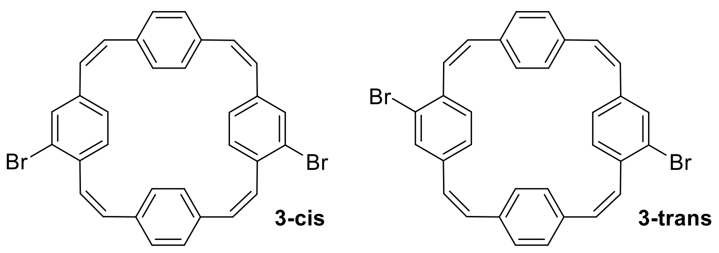


Molecular Weight: 566.34 Molecular Weight: 566.34

Prepared by modifying a procedure that was first reported for the synthesis of unsubstituted PCT.[3]

Compound **2** (8.20 g, 9.45 mmol, 1.0 equiv.) and terephthaldehyde (1.27 g, 9.45 mmol, 1.0 equiv.) were suspended in DMF (350 mL) and cooled to -40°C (using an acetonitrile/dry ice cooling bath). In a separate flask, LiOMe (1.08 g, 28.4 mmol, 3.0 equiv.) was dissolved in dry MeOH (40 mL). The LiOMe solution was added to the reaction mixture dropwise over the course of 8 hours (using a syringe pump) while maintaining a temperature of -40°C. After the addition, the solution was allowed to warm to room temperature and stirred overnight. The resulting bright yellow suspension was poured into stirring water (300 mL) and subsequently extracted with diethyl ether (4 x 200 mL). The organic phases were combined and washed with water (3 x 200 mL) and brine (200 mL). The solution was then dried over $MgSO_4$, filtered, and the solvent was removed *in vacuo* to yield a bright yellow solid. This was dissolved/suspended in $CH_2Cl_2$ (10 mL) and filtered over a pad of silica, eluting with $CH_2Cl_2$. The solvent was removed *in vacuo*, and the crude product was purified by recycling preparative GPC. The solvent was removed *in vacuo* to yield product **3** as a bright yellow powder containing a mixture of the two macrocyclic isomers **3-cis** and **3-trans** (240 mg, 0.42 mmol, 9%).

$^1$H NMR (400 MHz, $CDCl_3$): δ = 7.47-7.44 (m, 2H), 7.39-7.32 (m, 2H), 7.30-7.26 (m, 2H), 7.23-7.15 (m, 8H), 6.54-6.38 (m, 8H) ppm. $^{13}$C{$^1$H} NMR (101 MHz, $CDCl_3$): δ = 138.8, 138.4, 136.8, 136.5, 136.1, 135.7, 135.6, 135.4, 134.0, 133.6, 131.3, 131.1, 130.9, 130.8, 130.10, 130.08, 129.3, 129.2, 129.1, 129.0, 128.51, 128.45, 127.0, 126.9, 124.5, 124.2 ppm.

$^1$H NMR (400 MHz, $CD_2Cl_2$): δ = 7.475 (br s) *and* 7.467 (br s, combined 2H), 7.37 (d, $^3J_{H-H}$ = 8.0 Hz) *and* 7.35 (d, $^3J_{H-H}$ = 8.0 Hz, combined 2H), 7.32-7.26 (m, combined 2H), 7.21 (s) *and* 7.20 (s, combined 8H), 6.57- 6.41 (m, 8H) ppm. *Note: 'and' is used to denote signals which represent similar environments in the two different isomers. Here the integration was taken over both signals to allow for comparison with the ill-defined alkene signals.* $^{13}$C{$^1$H} NMR (101 MHz, $CD_2Cl_2$): δ = 139.3, 138.8, 137.1, 136.8, 136.4, 136.0, 135.9, 135.7, 134.2, 133.8, 131.4, 131.3, 131.14, 131.06, 130.4, 130.3, 129.44, 129.42, 129.35, 129.28, 129.24, 129.20, 128.69, 128.65, 127.3, 127.23, 124.6, 124.2 ppm.

*Note: In the $^{13}C$ NMR spectrum recorded in $CDCl_3$, two signals fewer than expected 28 signals were identified. These signals are believed to overlap with other signals due to the structural similarity of the two isomers. In the $^{13}C$ NMR spectrum recorded in $CD_2Cl_2$, the expected number of signals was identified.*

HRMS (*m/z*): $[M+H]^+$ calcd for $C_{32}H_{22}Br_2$: 565.0161; found: 565.0174 (APCI).

## 1.4. Dithienyl-substituted PCTs 4 (mixture of regioisomers 4-cis and 4-trans)

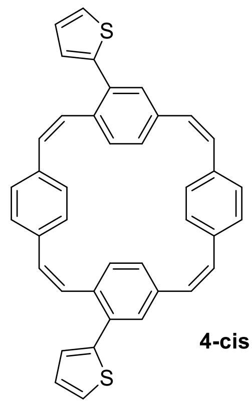

**4-cis**

Molecular Weight: 572.78

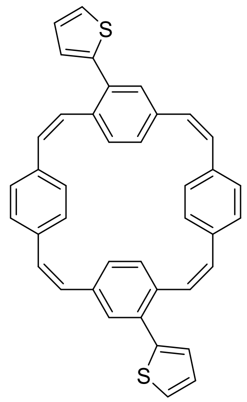

**4-trans**

Molecular Weight: 572.78

Dibrominated PCT **3** (50 mg, 0.088 mmol, 1.0 equiv.), thiophene-2-boronic acid pinacol ester (74 mg, 0.353 mmol, 4.0 equiv.), $Na_2CO_3$ (94 mg, 0.88 mmol, 10 equiv.) and $Pd(PPh_3)_4$ (20 mg, 0.018 mmol, 0.2 equiv.) were suspended in a mixture of toluene (15 mL), EtOH (5 mL) and deionised water (5 mL) under $N_2$ atmosphere. The mixture was stirred vigorously and sparged with $N_2$ for 1 hour. It was then heated to 80°C and stirred for 2 days. After cooling to room temperature, saturated aqueous $NH_4Cl$ solution (100 mL) was added, and the mixture was extracted with $CH_2Cl_2$ (3 x 100 mL). The organic phases were combined, washed with deionised water (3 x 100 mL) and brine (100 mL), dried over $MgSO_4$, filtered, and the solvent was removed *in vacuo*. Following this, the product was purified by recycling preparative GPC. This gave product **4** as a mixture of the two isomers **4-cis** and **4-trans** (40 mg, 0.070 mmol, 79%).

$^1H$ NMR (400 MHz, $CDCl_3$): δ = 7.50-7.16 and 7.08-7.01 (both m, combined 20H), 6.57-6.37 (m, 8H) ppm.

$^1H$ NMR (400 MHz, $CD_2Cl_2$): δ = 7.53-7.17 (m, 18H), 7.09-7.02 (m, 2H), 6.59-6.38 (m, 8H) ppm. $^{13}C\{^1H\}$ NMR (101 MHz, $CD_2Cl_2$): δ = 142.6, 137.27, 137.25, 136.6, 136.0, 135.4, 134.3, 131.5, 134.4, 130.9, 130.82, 130.77, 130.6, 130.24, 130.16, 130.09, 130.06, 130.04, 129.7, 129.40, 129.35, 129.30, 129.25, 129.22, 129.18, 129.1, 127.74, 127.68, 127.66, 127.62, 127.58, 127.5, 126.2, 126.13 ppm. *Note: In the*

*$^{13}$C NMR spectrum, two product signals fewer than expected 28 signals were identified. These signals are believed to overlap with other signals due to the structural similarity of the two isomers.*

HRMS (*m/z*): $[M+H]^+$ calcd for $C_{40}H_{28}S_2$: 573.1705; found: 573.1703 (APCI).

### 1.5. Bis(bithiophene)-substituted PCTs 5 (mixture of regioisomers 5-cis and 5-trans)

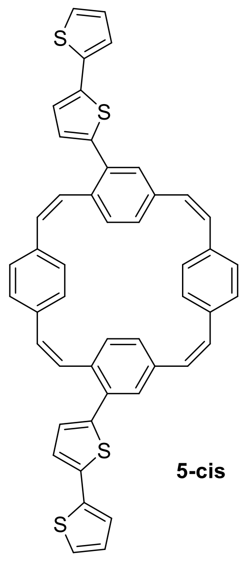

**5-cis**

Molecular Weight: 737.02

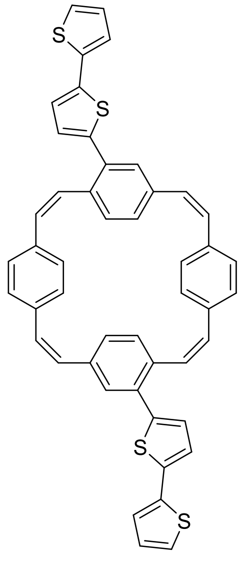

**5-trans**

Molecular Weight: 737.02

Compound **3** (100 mg, 0.177 mmol, 1.0 equiv.), 2,2'-bithiophene-5-boronic acid pinacol ester (155 mg, 0.530 mmol, 3.0 equiv.), $Na_2CO_3$ (188 mg, 1.77 mmol, 10.0 equiv.) and $Pd(PPh_3)_4$ (20 mg, 0.018 mmol, 0.1 equiv.) were suspended in a mixture of toluene (30 mL), EtOH (10 mL) and deionised water (10 mL) under $N_2$ atmosphere. The mixture was stirred vigorously and sparged with $N_2$ for 1 hour. It was then heated to 80°C and stirred for 2 days. After cooling to room temperature, saturated aqueous $NaHCO_3$ solution (100 mL) was added, and the mixture was extracted with $CH_2Cl_2$ (3 x 100 mL). The organic phases were combined, washed with deionised water (3 x 200 mL) and brine (150 mL), dried over $MgSO_4$, filtered, and the solvent was removed *in vacuo*. Following this, the product was purified by recycling preparative GPC to yield product **5** as a mixture of the two isomers **5-cis** and **5-trans** (42 mg, 0.057 mmol, 32%).

$^1H$ NMR (400 MHz, $CDCl_3$): δ = 7.49-7.42 (m, 2H), 7.42-7.38 (m, 2H), 7.36-7.28 (m, 6H), 7.25 (s, 4H), 7.22-7.19 (m, 2H), 7.19-7.14 (m, 2H), 7.14-7.06 (m, 4H), 7.03-6.98 (m, 2H), 6.60-6.43 (m, 8H) ppm. $^{13}C\{^1H\}$ NMR (101 MHz, $CDCl_3$): δ = 141.2, 137.9, 137.8, 137.49, 137.46, 137.0, 136.2, 135.9, 135.8, 135.7, 134.9, 134.6, 134.1, 133.8, 130.8, 130.7, 130.6, 130.3, 130.2, 130.1, 130.0, 129.9, 129.5, 129.4, 129.20, 129.15, 129.1, 128.2, 128.1, 128.0, 127.5, 124.54 124.52, 124.13, 124.10, 123.8 ppm. *Note: In the $^{13}C$ NMR spectrum, 8 of the 44 signals are considered to overlap with other signals due to the structural similarity of the two isomers.*

$^1$H NMR (400 MHz, $CD_2Cl_2$): δ = 7.45-7.39 (m, 4H), 7.35-7.21 (m, 12H), 7.20-7.16 (m, 2H), 7.15-7.07 (m, 4fH), 7.05-7.00 (m, 2H), 6.63-6.46 (m, 8H) ppm.

MS (*m/z*): $[M]^+$ calcd for $C_{48}H_{32}S_4$: 736.14; found: 736.64 (MALDI).

### 1.6. Bis(3-hexylthienyl)-substituted PCT isomers 6-cis and 6-trans (separated regioisomers)

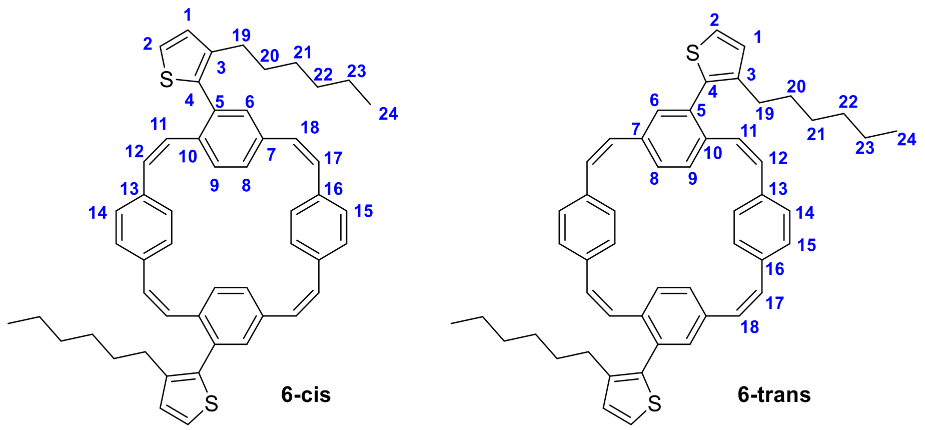


Molecular Weight: 741.11 Molecular Weight: 741.11

Compound **3** (50 mg, 0.088 mmol, 1.0 equiv), 3-hexylthiophene-2-boronic acid pinacol ester (78 mg, 0.265 mmol, 3.0 equiv.), $Na_2CO_3$ (94 mg, 0.88 mmol, 10 equiv.) and $Pd(PPh_3)_4$ (20 mg, 0.018 mmol, 0.2 equiv.) were suspended in a mixture of toluene (40 mL), EtOH (7 mL) and deionised water (7 mL) under $N_2$ atmosphere. The mixture was stirred vigorously and sparged with $N_2$ for 1 hour. It was then heated to 80°C and stirred overnight. After cooling to room temperature, saturated aqueous $NH_4Cl$ solution (50 mL) was added, and the mixture was extracted with $CH_2Cl_2$ (3 x 50 mL). The organic phases were combined, dried over $MgSO_4$, filtered, and the solvent was removed *in vacuo*. The crude product was dissolved in $CH_2Cl_2$, filtered over a short pad of silica, eluting with $CH_2Cl_2$, and the solvent was again removed *in vacuo*. Following this, the mixture was purified by recycling preparative GPC yielding two separate product fractions which, upon removal of the solvent, presented as bright yellow solids. Each of the fractions contained a different isomer of the cross-coupling product; isomer **6-trans** (28 mg, 0.04 mmol, 43%) eluted first, followed by isomer **6-cis** (22 mg, 0.03 mmol, 34%).

**6-cis**:

$^1$H NMR (400 MHz, $CDCl_3$): δ = 7.60 (d, *J* = 8.0 Hz, 2H, *H*9), 7.50 (dd, *J* = 8.0, 1.2 Hz, 2H, *H*8), 7.35 (s, 4H, *H*14/*H*15), 7.34 (s, 4H, *H*14/*H*15), 7.25 (d, *J* = 5.2 Hz, 2H, *H*2), 7.18 (d, *J* = 1.6 Hz, 2H, *H*6), 6.96 (d, *J* = 5.2 Hz, 2H, *H*1), 6.48 (d, *J* = 12.4 Hz, 2H, *H*11/*H*12), 6.43 (d, *J* = 12.4 Hz, 2H, *H*11/*H*12), 6.31 (d, *J* = 12.4 Hz, 2H, *H*17/*H*18), 6.18 (d, *J* = 12.4 Hz, 2H, *H*17/*H*18), 2.47 (t, *J* = 7.6 Hz, 4H, *H*19), 1.51 (quin, *J* = 7.6 Hz, 4H, *H*20), 1.24-1.07 (m, 12H, *H*21, *H*22, *H*23), 0.78 (t, $J_{H\text{-}H}$ = 6.8 Hz, 6H, *H*24) ppm. $^{13}C\{^1H\}$ NMR (101 MHz, $CDCl_3$): δ = 140.9 (*C*4), 136.7 (*C*7), 136.6 (*C*13/*C*16), 136.02 (*C*10), 136.00 (*C*3), 135.7 (*C*13/*C*16),

135.1 (*C*5), 133.2 (*C*6), 130.3 (*C*11 and *C*12), 129.7 (*C*17/*C*18), 129.6 (*C*17/*C*18), 129.2 (*C*14/*C*15), 129.1 (*C*14/*C*15), 128.9 (*C*9), 128.7 (*C*1), 127.4 (*C*8), 124.4 (*C*2), 31.7 (*C*21/*C*22/*C*23), 30.5 (*C*20), 29.2 (*C*21/*C*22/*C*23), 28.9 (*C*19), 22.7 (*C*21/*C*22/*C*23), 14.2 (*C*24) ppm.

HRMS (*m/z*): $[M+H]^+$ calcd for $C_{52}H_{52}S_2$: 741.3583; found: 741.3586 (APCI).

**6-trans**:

$^1$H NMR (400 MHz, $CDCl_3$): δ = 7.60 (d, *J* = 8.0 Hz, 2H, *H*9), 7.53 (dd, *J* = 8.4, 1.6 Hz, 2H, *H*8), 7.40 (d, *J* = 8.4 Hz, 4H, *H*14/*H*15), 7.35 (d, *J* = 8.4 Hz, 4H, *H*14/*H*15), 7.26 (d, *J* = 5.2 Hz, 2H, *H*2), 7.20 (d, *J* = 1.6 Hz, 2H, *H*6), 6.97 (d, *J* = 5.2 Hz, 2H, *H*1), 6.49-6.41 (m, 4H, *H*11/*H*12), 6.31 (d, *J* = 12.4 Hz, 2H, *H*18), 6.19 (d, *J* = 12.4 Hz, 2H, *H*17), 2.47 (t, *J* = 7.6 Hz, 4H, *H*19), 1.52 (quin, *J* = 7.6 Hz, 4H, *H*20), 1.23-1.08 (m, 12H, *H*21, *H*22, *H*23), 0.78 (t, *J* = 7.8 Hz, 6H, *H*24) ppm. $^{13}$C{$^1$H} NMR (101 MHz, $CDCl_3$): δ = 140.9 (*C*4), 136.9 (*C*7), 136.3 (*C*13/ *C*16), 136.2 (*C*10), 135.9 (*C*3), 135.6 (*C*13/*C*16), 134.9 (*C*5), 133.6 (*C*6), 130.4 (*C*11/*C*12), 129.6 (*C*11/*C*12), 129.5 (*C*18), 129.3 (*C*17), 129.2 (*C*14/*C*15), 129.0 (*C*14/*C*15), 128.9 (*C*9), 128.6 (*C*1), 127.2 (*C*8), 124.3 (*C*2), 31.7 (*C*21/*C*22/*C*23), 30.5 (*C*20), 29.1 (*C*21/*C*22/*C*23), 28.9 (*C*19), 22.6 (*C*21/*C*22/*C*23), 14.2 (*C*24) ppm.

HRMS (*m/z*): $[M+H]^+$ calcd for $C_{52}H_{52}S_2$: 741.3583; found: 741.3563 (APCI).

## 2. $^{1}H$ and $^{13}C\{^{1}H\}$ NMR spectra

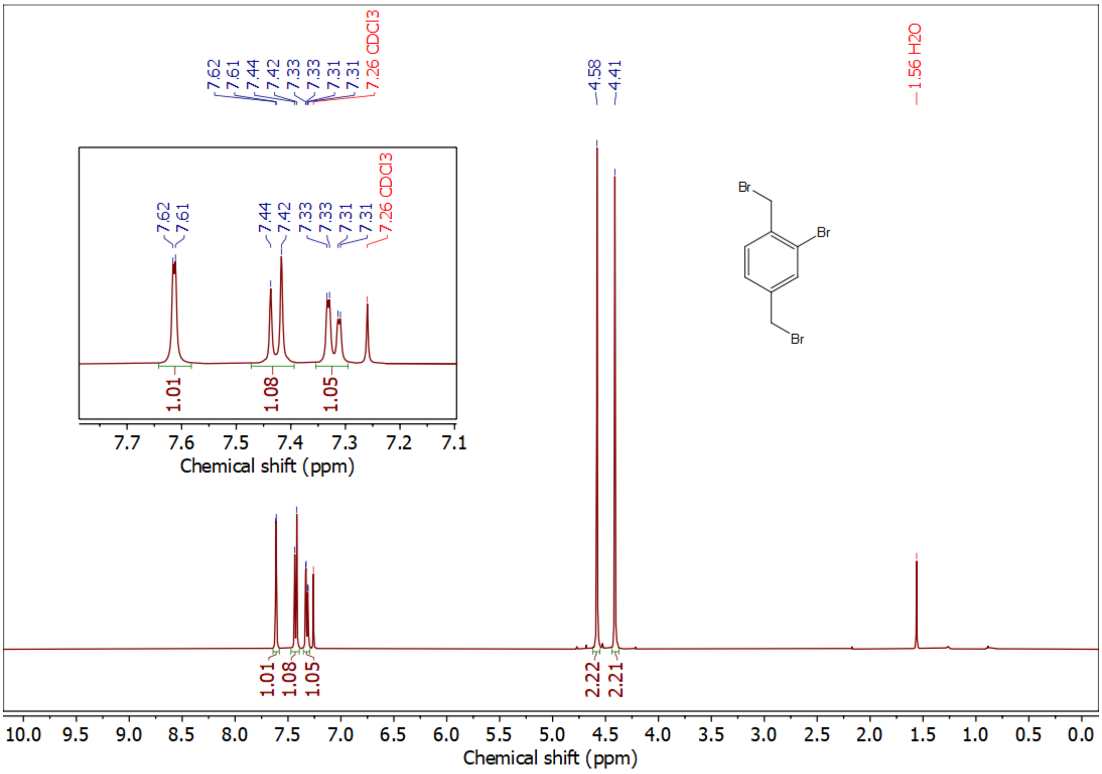


**Figure S1.** $^{1}H$ NMR (400 MHz, $CDCl_3$) of compound **1**.

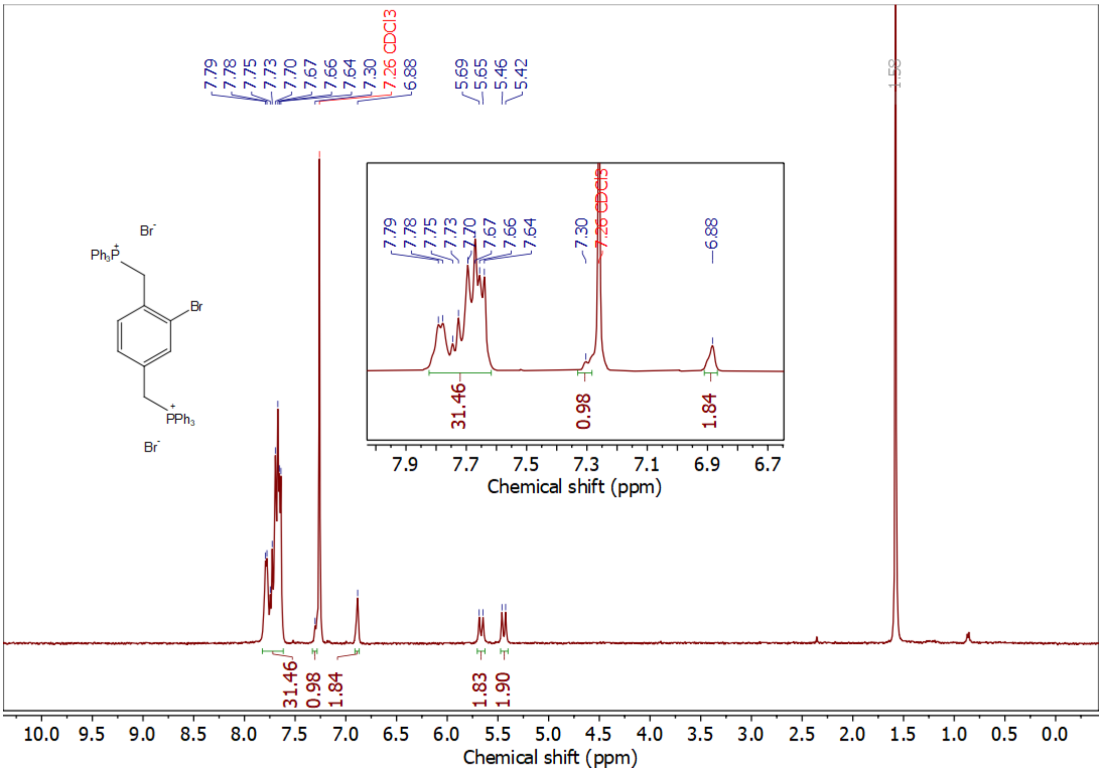


**Figure S2.** $^{1}H$ NMR (400 MHz, $CDCl_3$) of compound **2**.

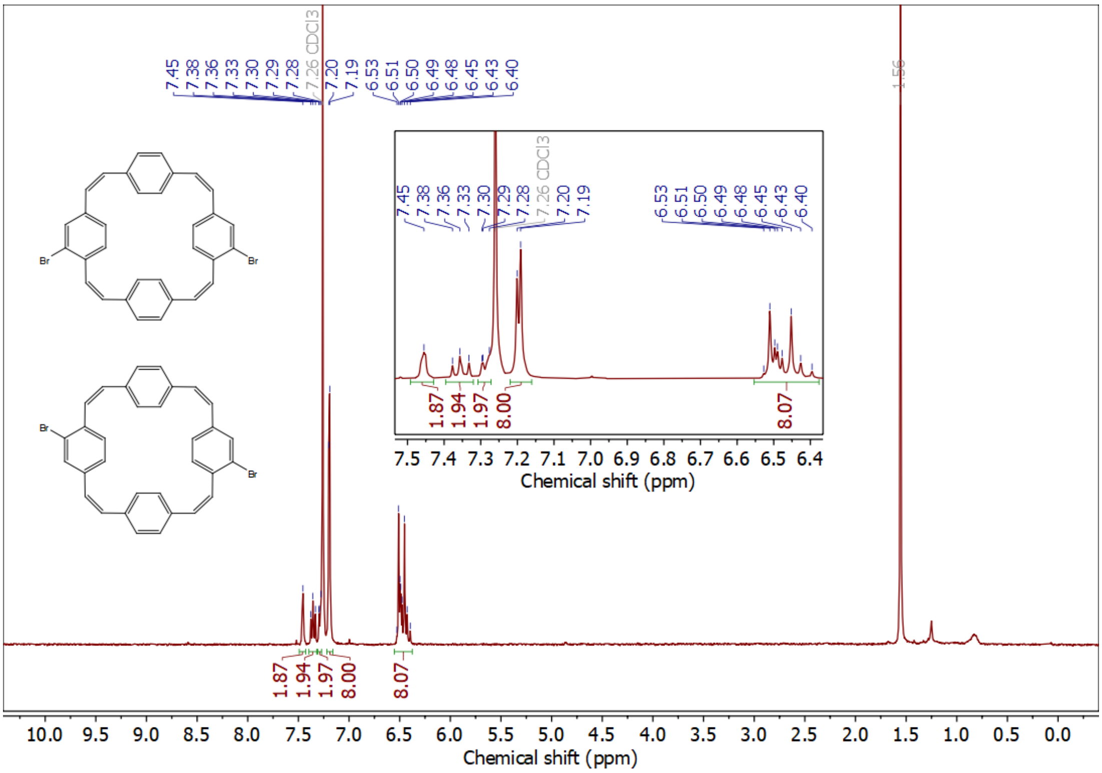


**Figure S3.** $^{1}H$ NMR (400 MHz, $CDCl_3$) of compounds **3**.

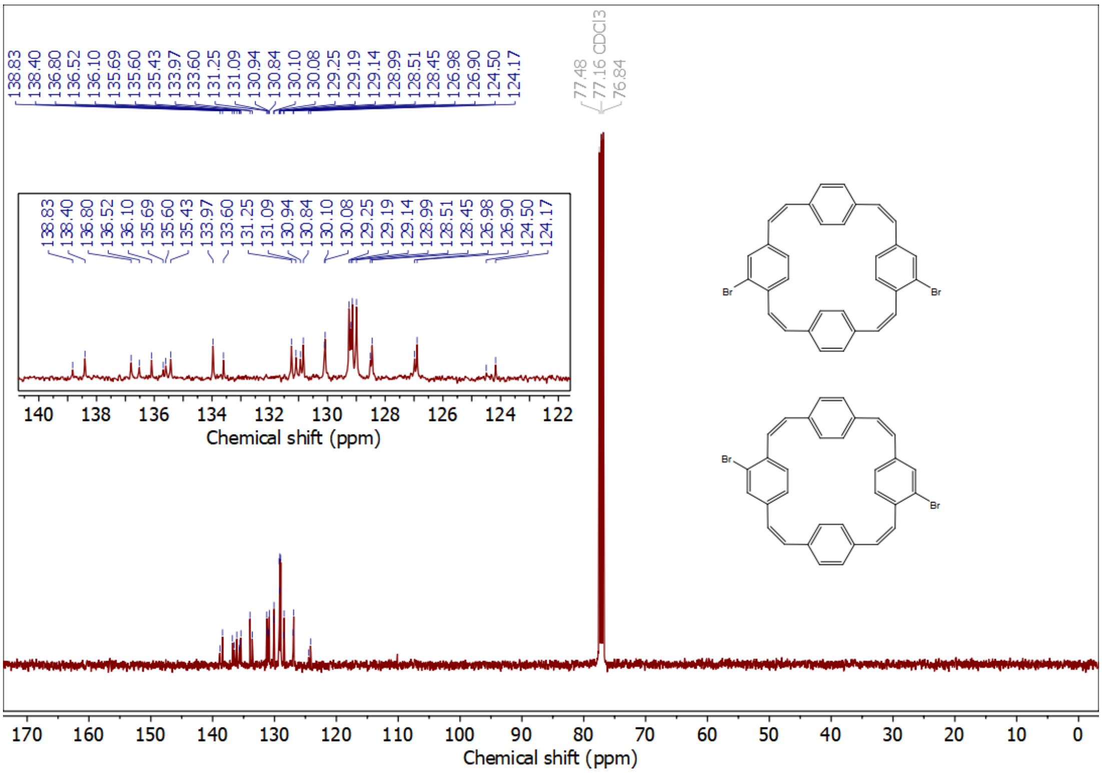


**Figure S4.** $^{13}C\{^{1}H\}$ NMR (101 MHz, $CDCl_3$) of compounds **3**.

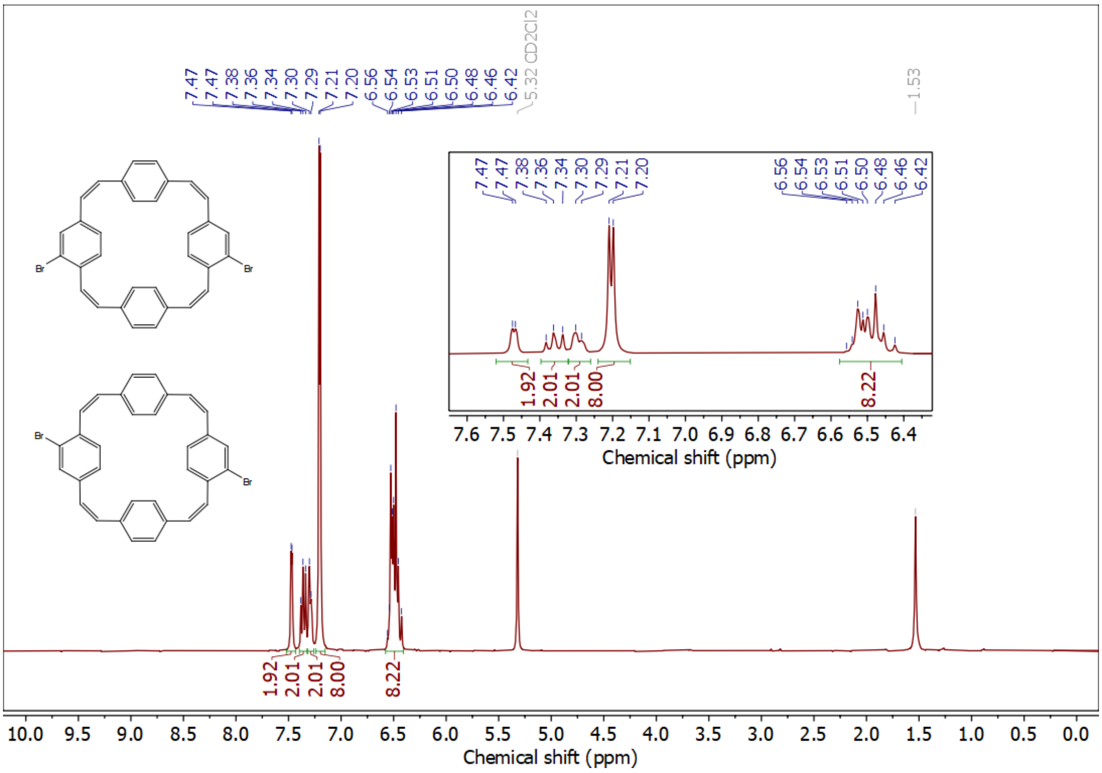

**Figure S5.** $^{1}H$ NMR (400 MHz, $CD_2Cl_2$) of compounds **3**.

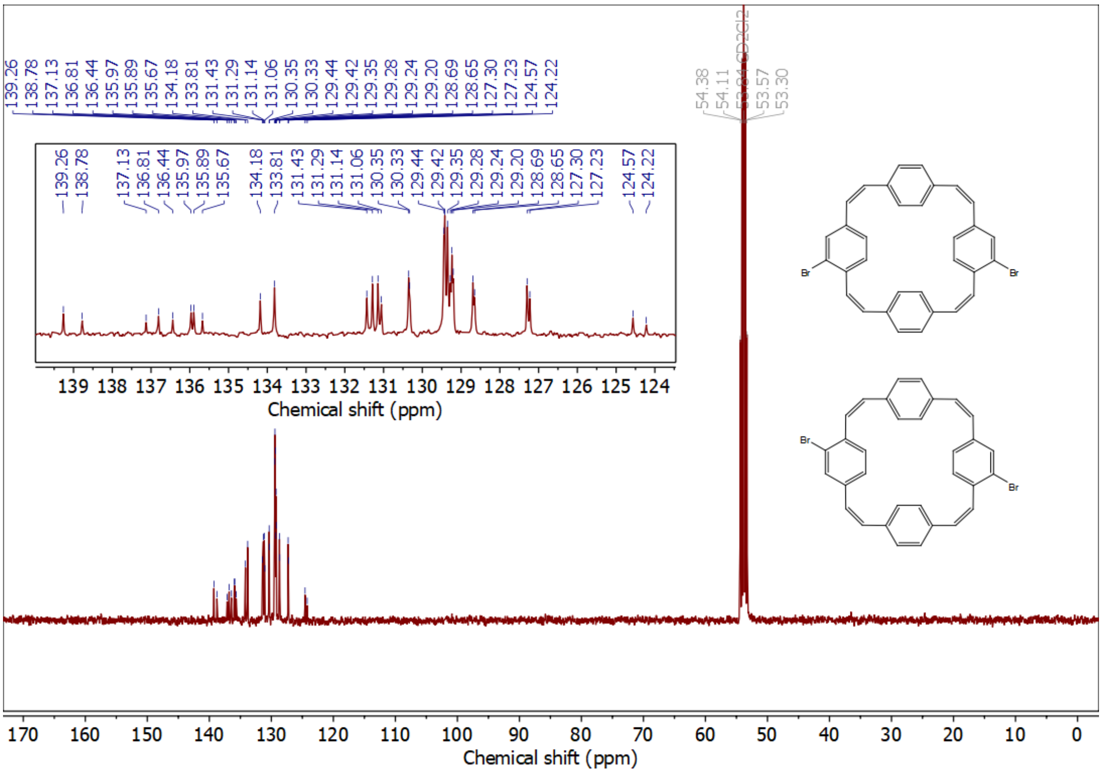

**Figure S6.** $^{13}C\{^{1}H\}$ NMR (101 MHz, $CD_2Cl_2$) of compounds **3**.

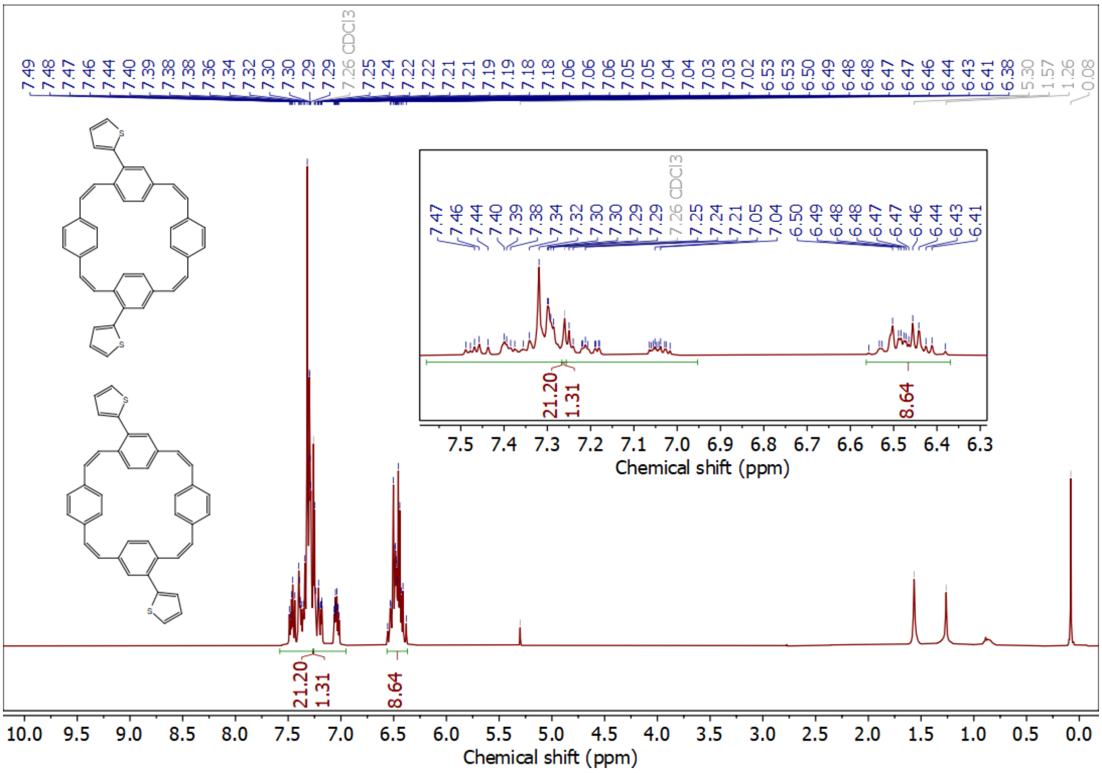

**Figure S7.** $^{1}$H NMR (400 MHz, $CDCl_3$) of compounds **4**.

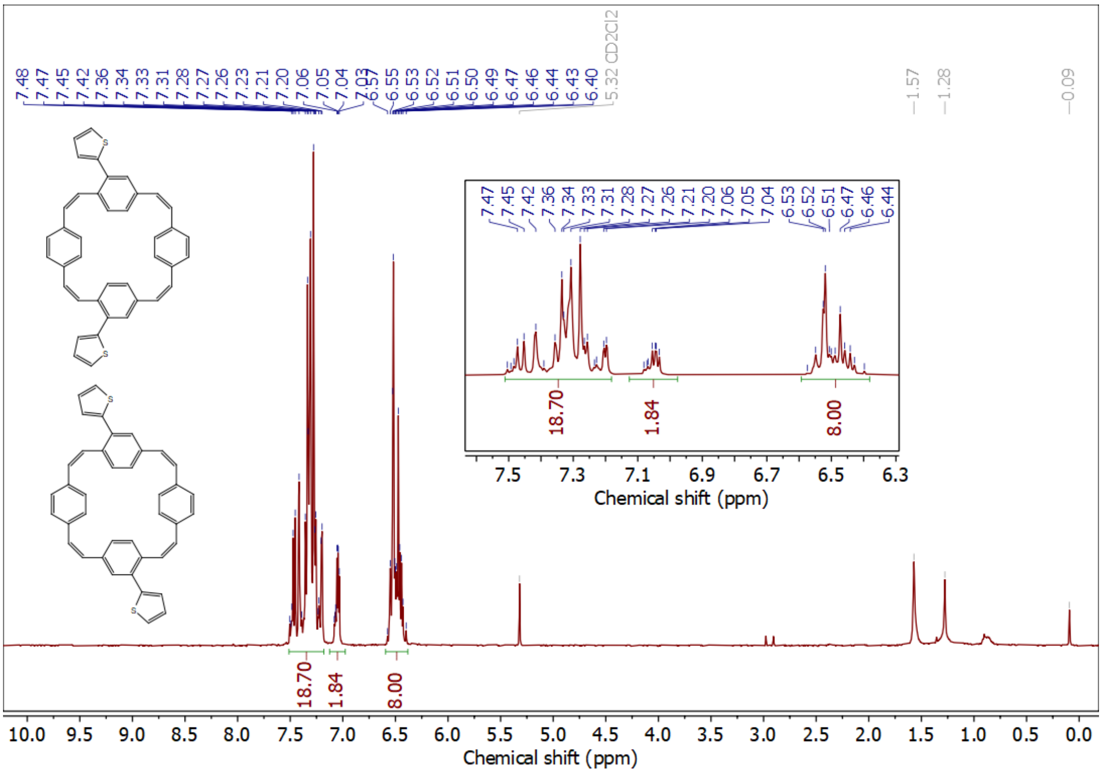

**Figure S8.** $^{1}$H NMR (400 MHz, $CD_2Cl_2$) of compounds **4**.

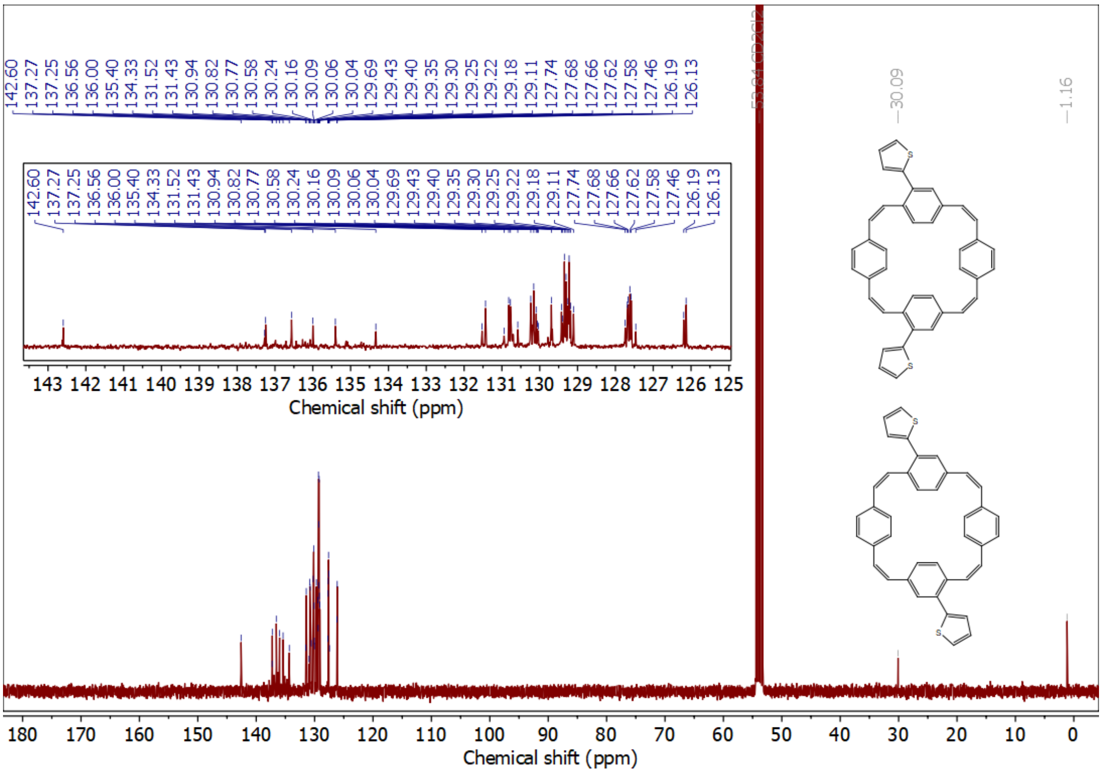


**Figure S9.** $^{13}C\{^1H\}$ NMR (101 MHz, $CD_2Cl_3$) of compounds **4**.

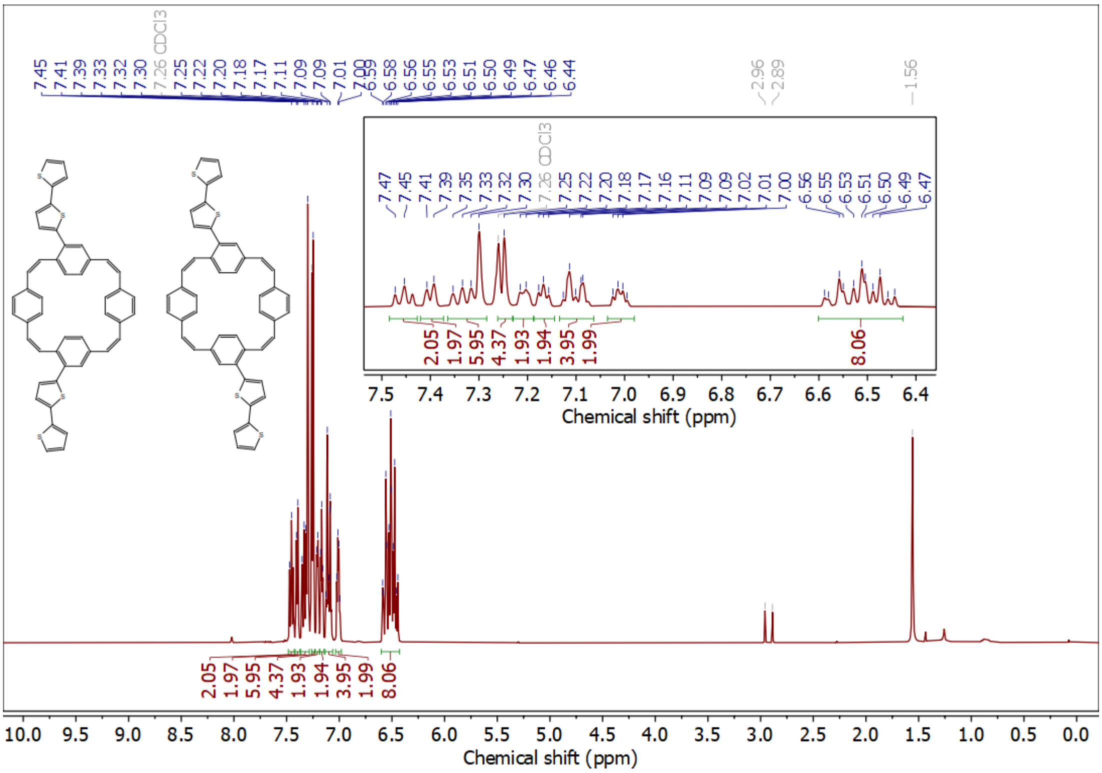


**Figure S10.** $^1H$ NMR (400 MHz, $CDCl_3$) of compounds **5**.

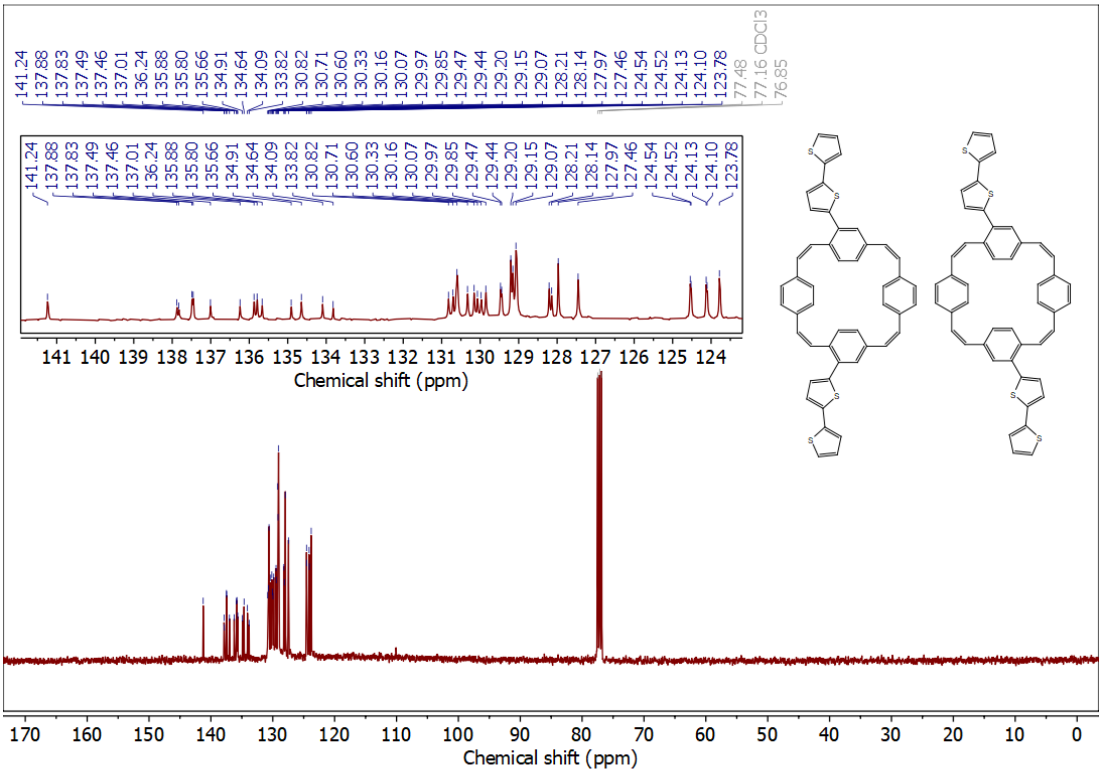


**Figure S11.** $^{13}C\{^{1}H\}$ NMR (101 MHz, $CDCl_3$) of compounds **5**.

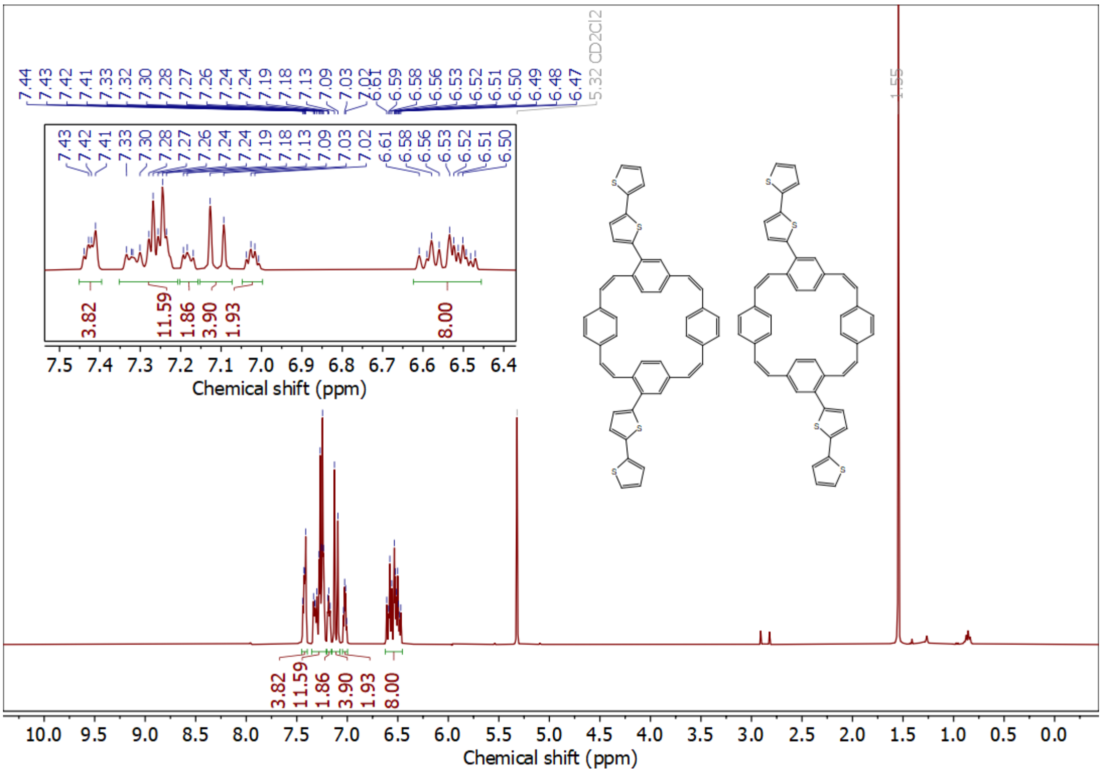


**Figure S12.** $^{1}H$ NMR (400 MHz, $CD_2Cl_2$) of compounds **5**.

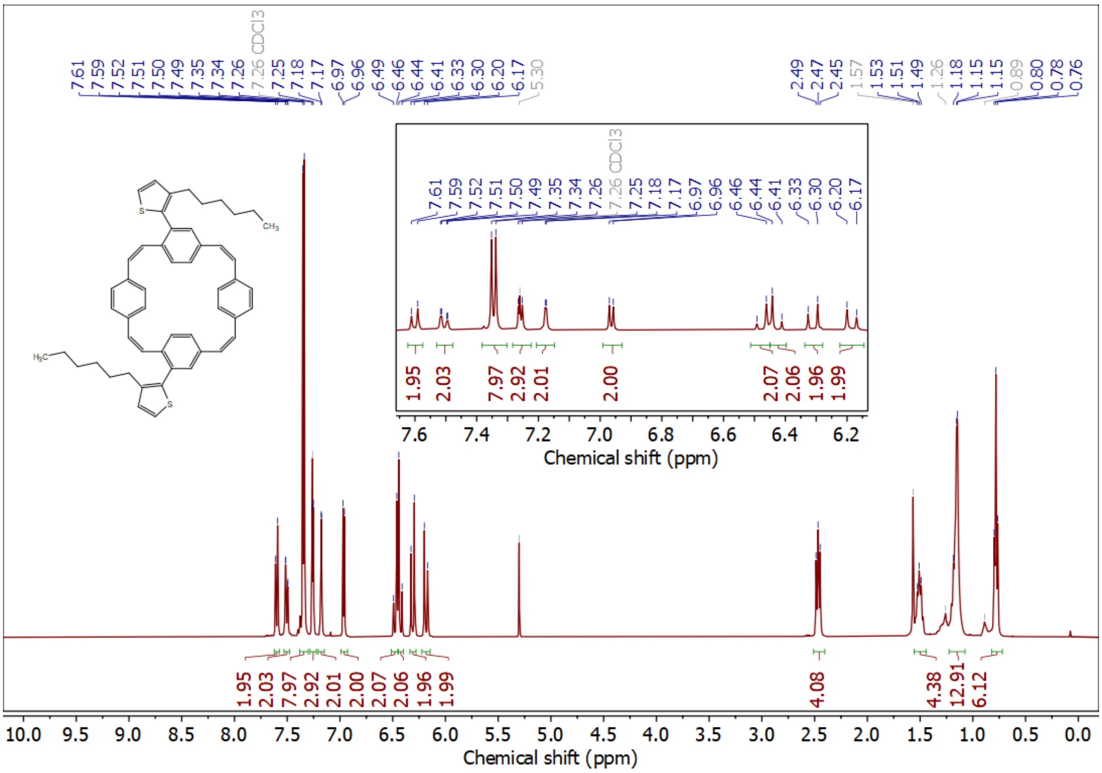


**Figure S13.** $^{1}$H-NMR (400 MHz, $CDCl_3$) of compound **6-cis**.

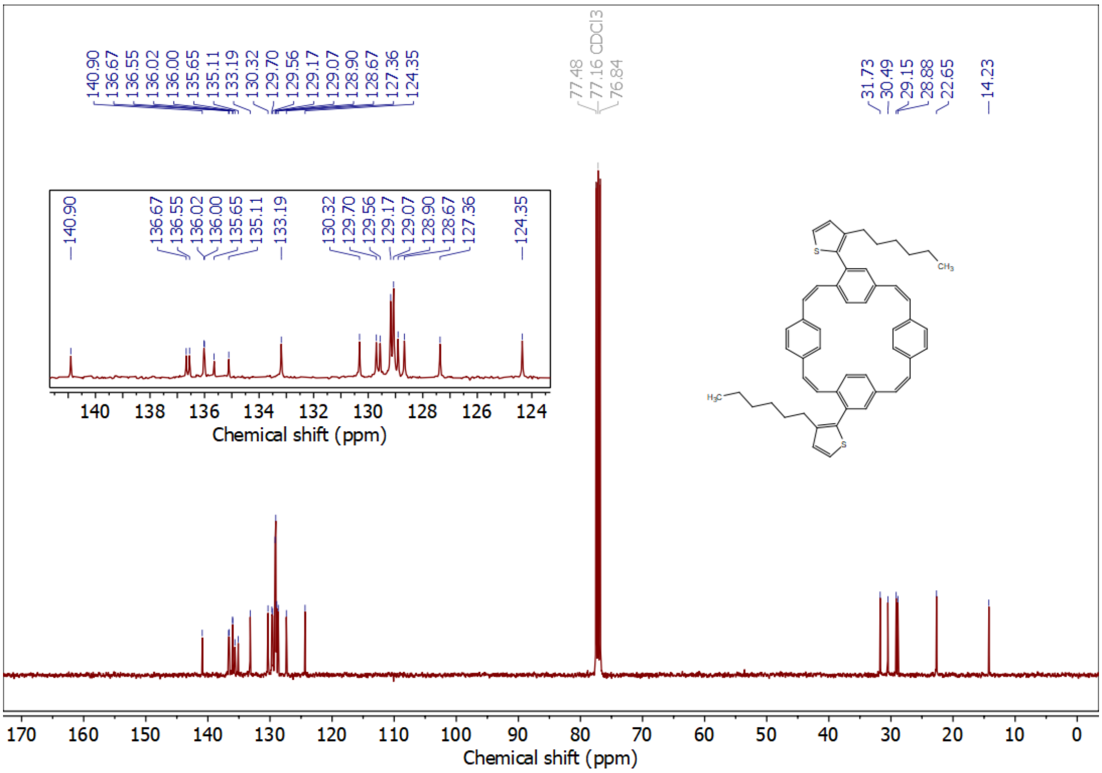


**Figure S14.** $^{13}C\{^{1}H\}$ NMR (101 MHz, $CDCl_3$) of compound **6-cis**.

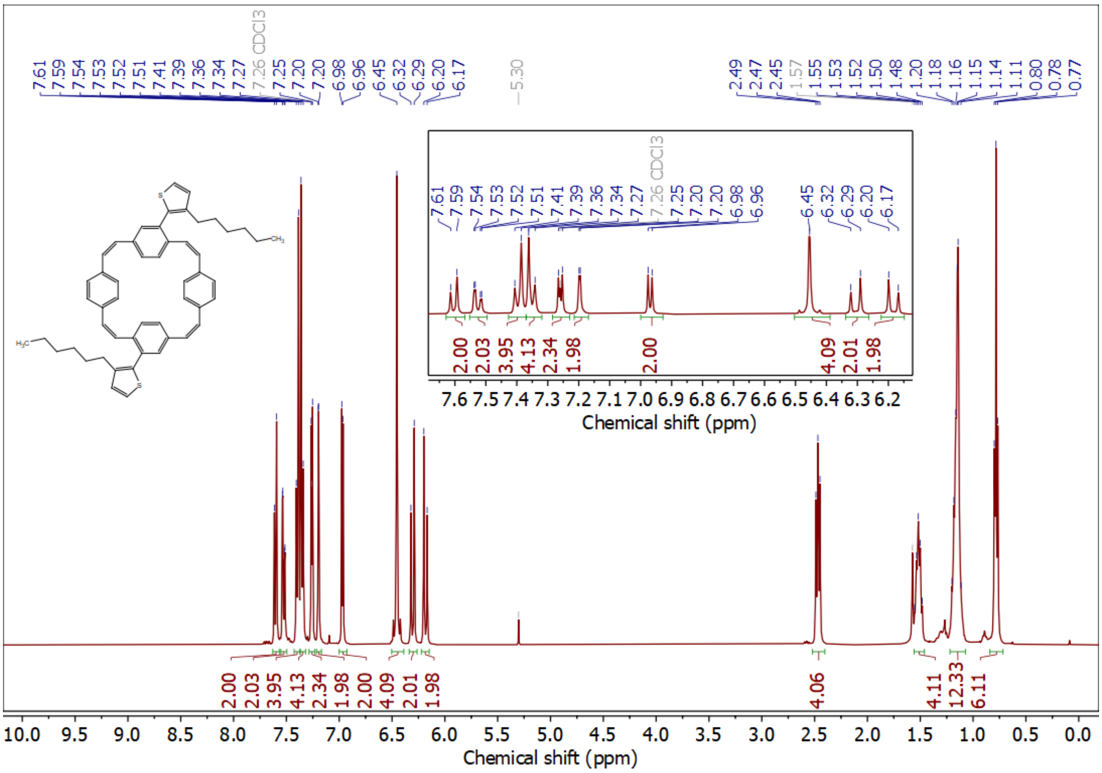

**Figure S15.** $^{1}H$ NMR (400 MHz, $CDCl_3$) of compound **6-trans**.

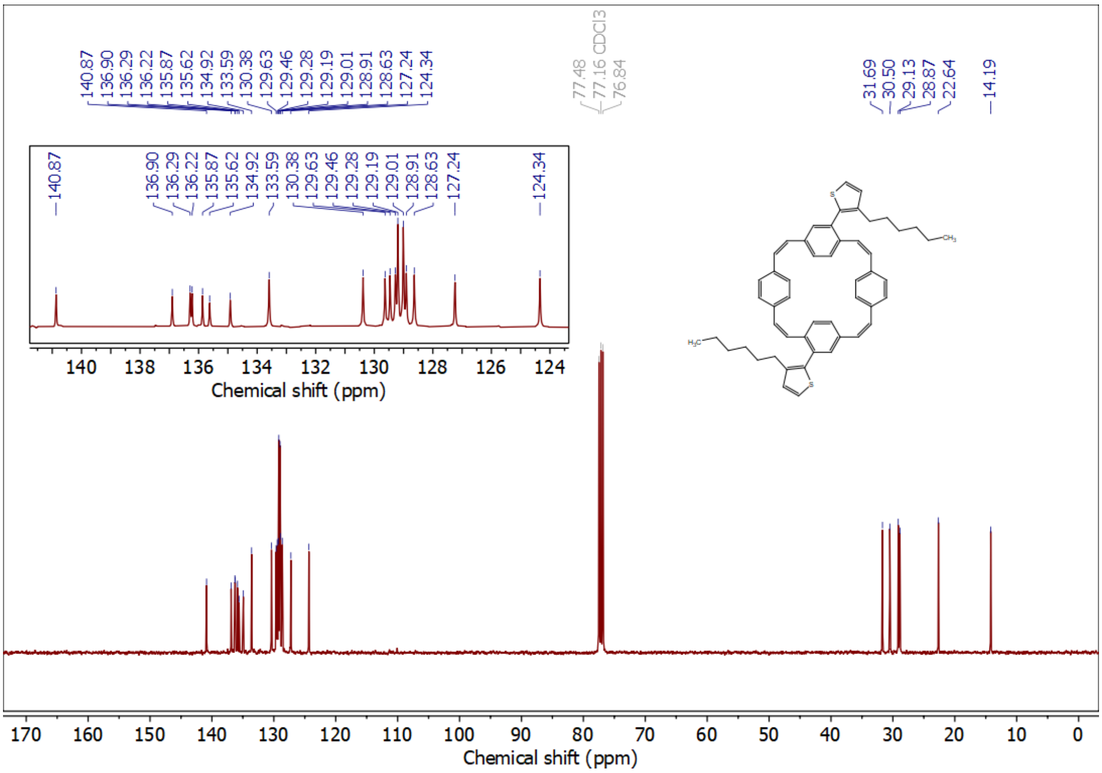

**Figure S16.** $^{13}C\{^{1}H\}$ NMR (101 MHz, $CDCl_3$) of compound **6-trans**.

# 3. Mass spectra

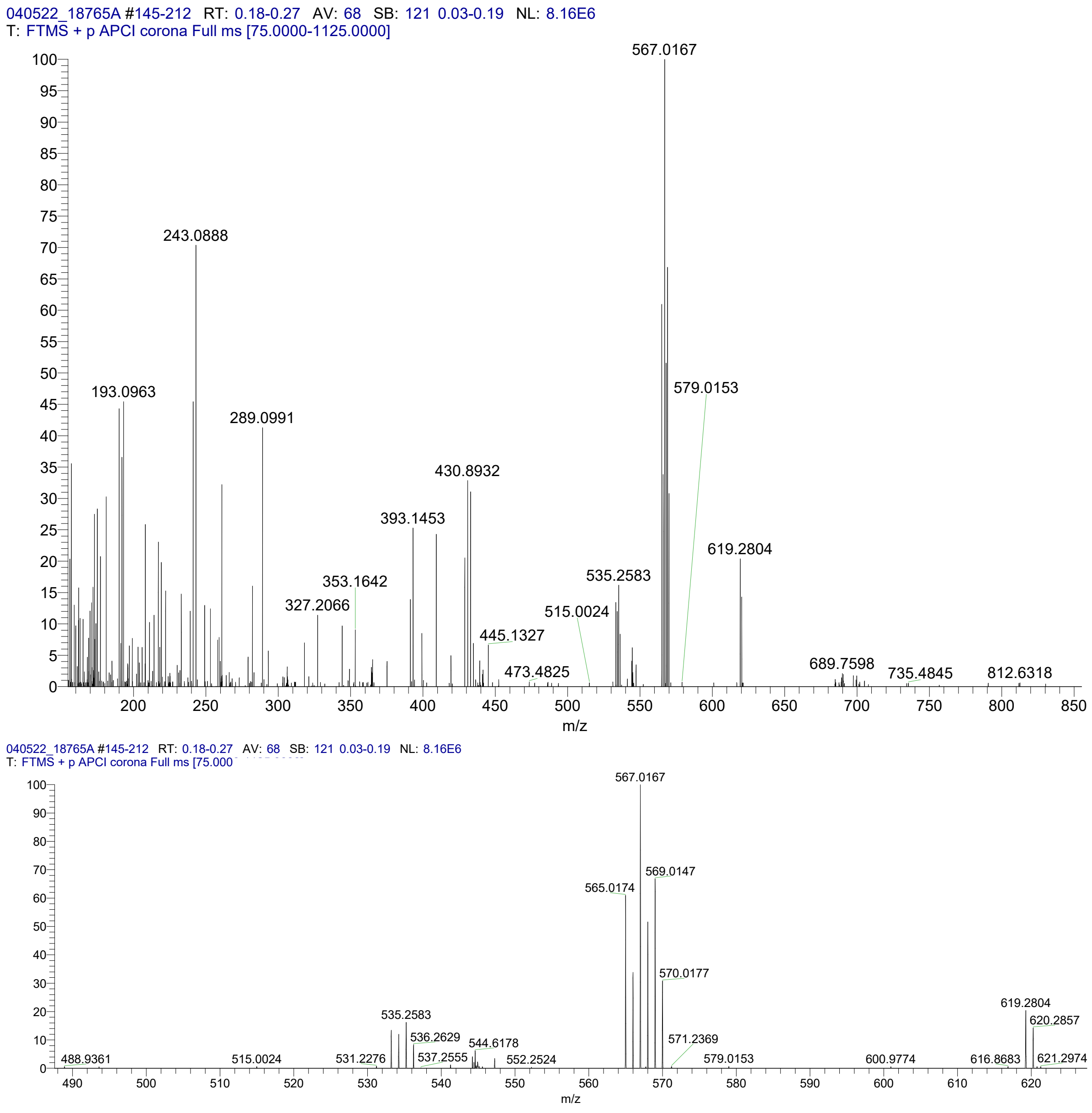


**Figure S17.** HRMS (APCI) spectra of compounds **3**.

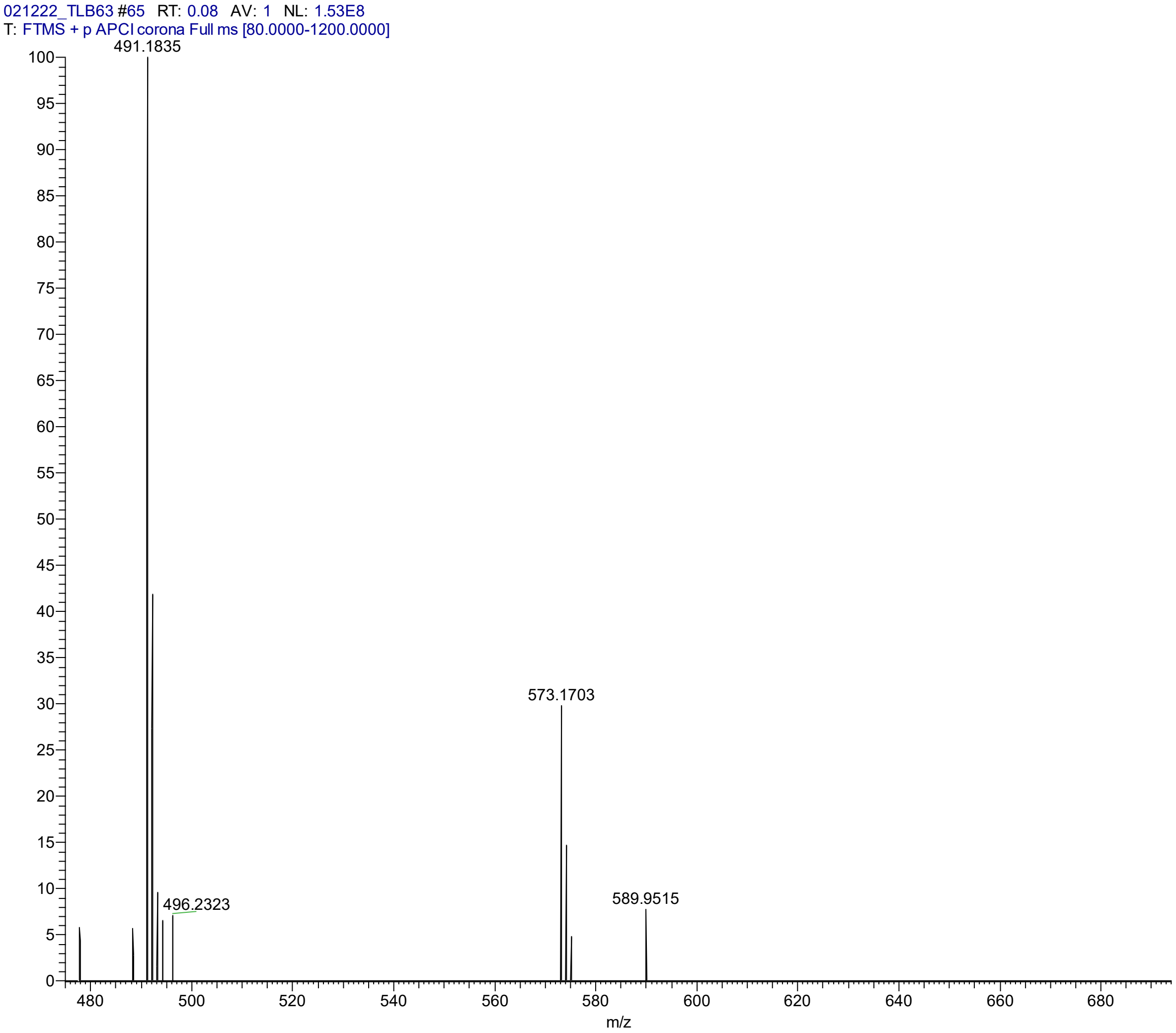


**Figure S18.** HRMS (APCI) spectrum of compounds **4**.

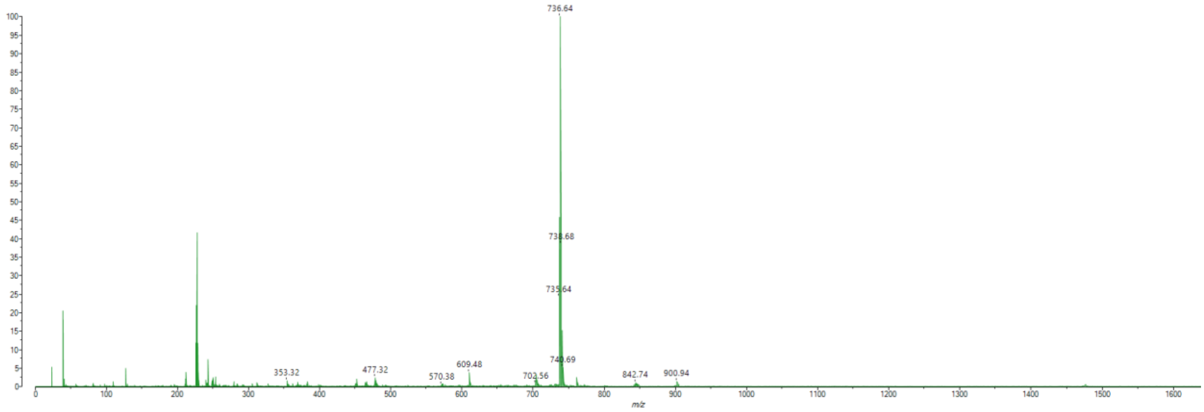


**Figure S19.** MS (MALDI) spectrum of compounds **5**.

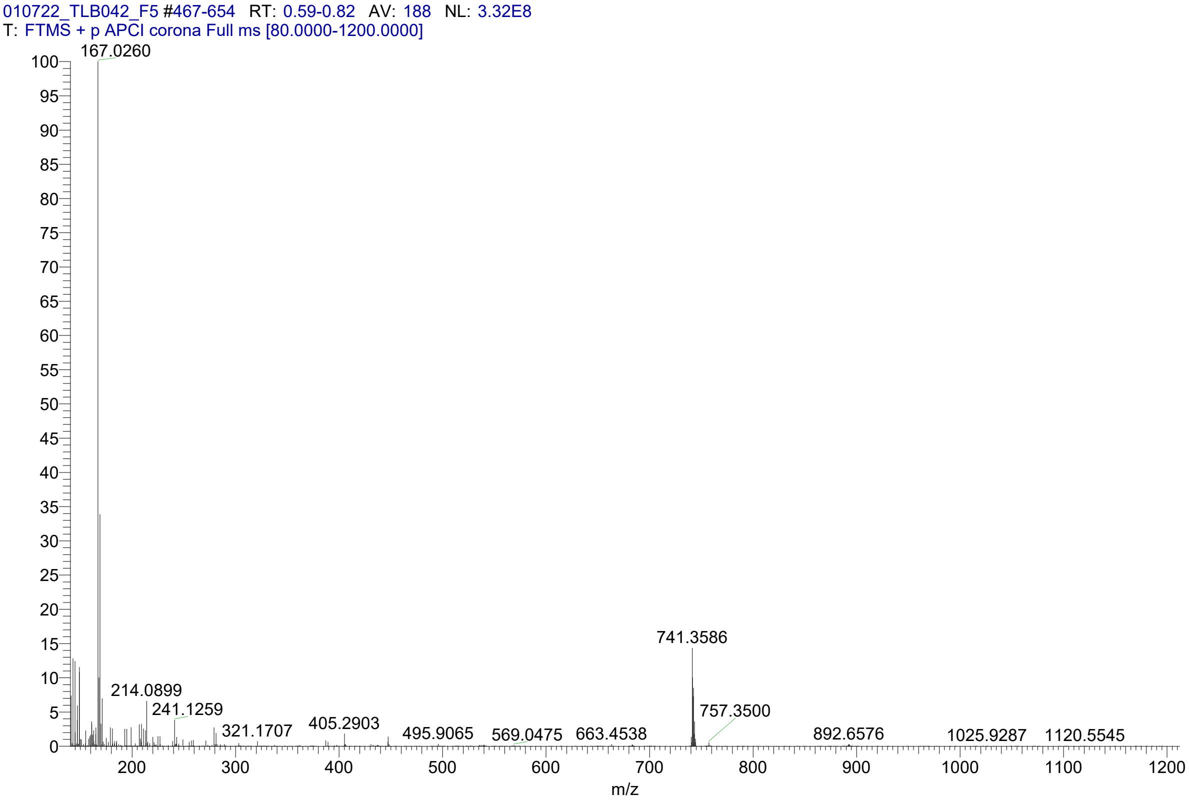


**Figure S20.** HRMS (APCI) spectrum of compounds **6-cis**.

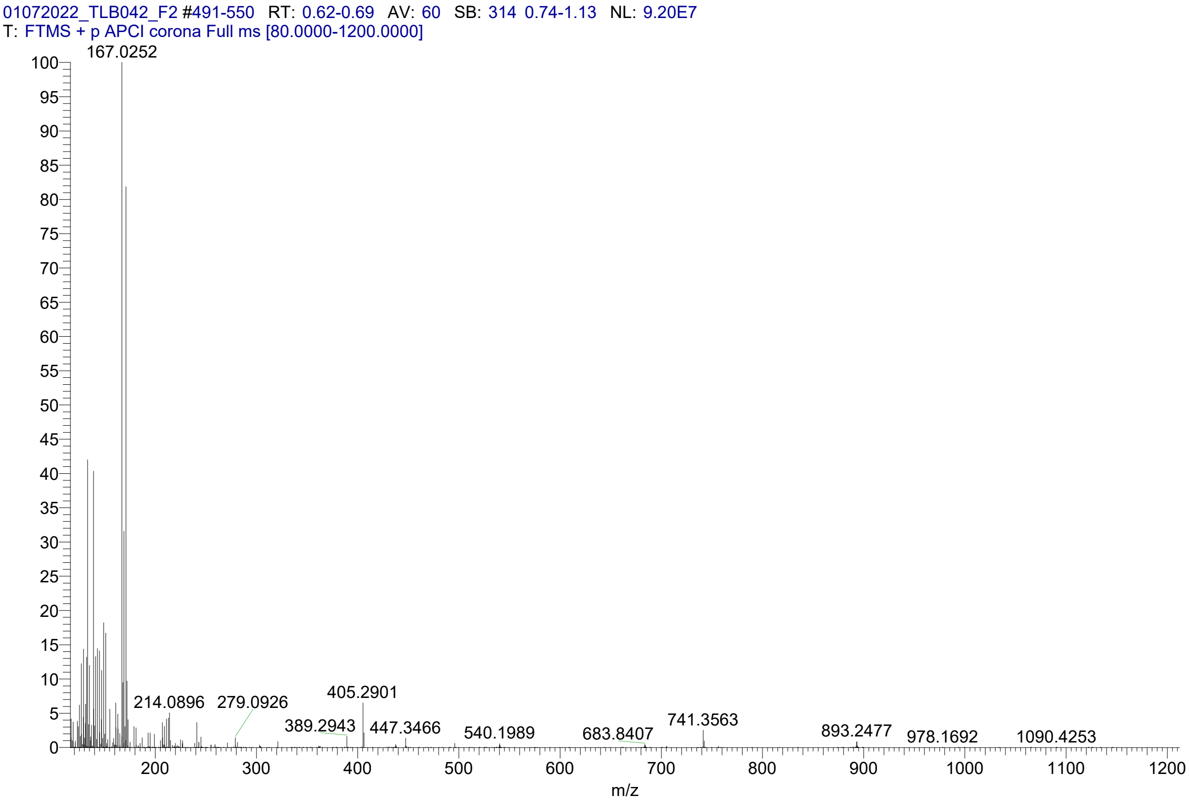


**Figure S21.** HRMS (APCI) spectrum of compounds **6-trans**.

## 4. Thermogravimetric analysis (TGA)

TGA was carried out at a heating rate of 10°C $min^{-1}$ under a nitrogen flow of 50 mL $min^{-1}$. The initial weight loss of **6-cis** was attributed to $CH_2Cl_2$ inclusions (presumably a result of the porous nature of the macrocyclic material), which cannot easily be removed following purification by recycling GPC.

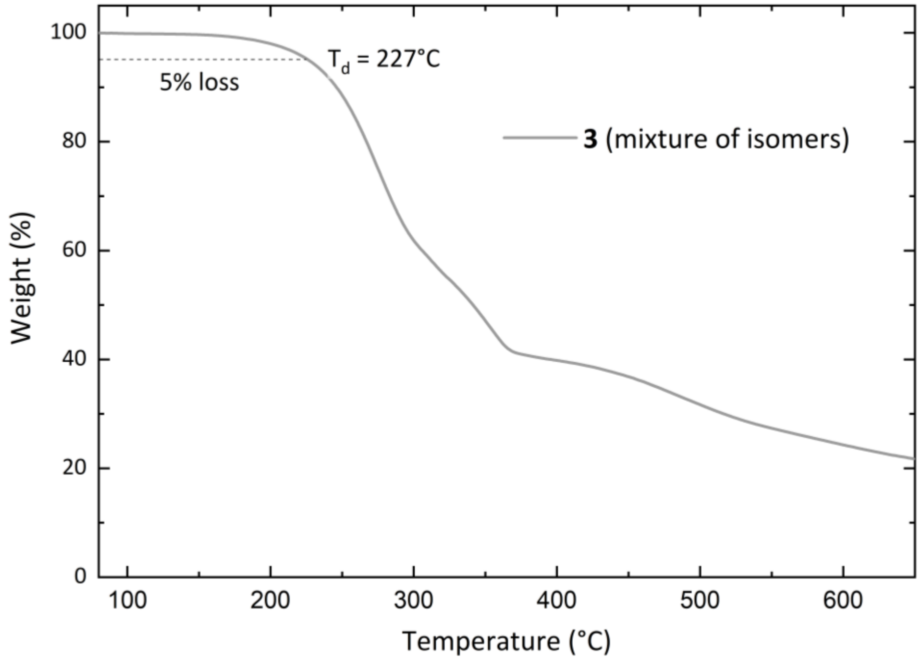


**Figure S22.** TGA of compounds **3**.

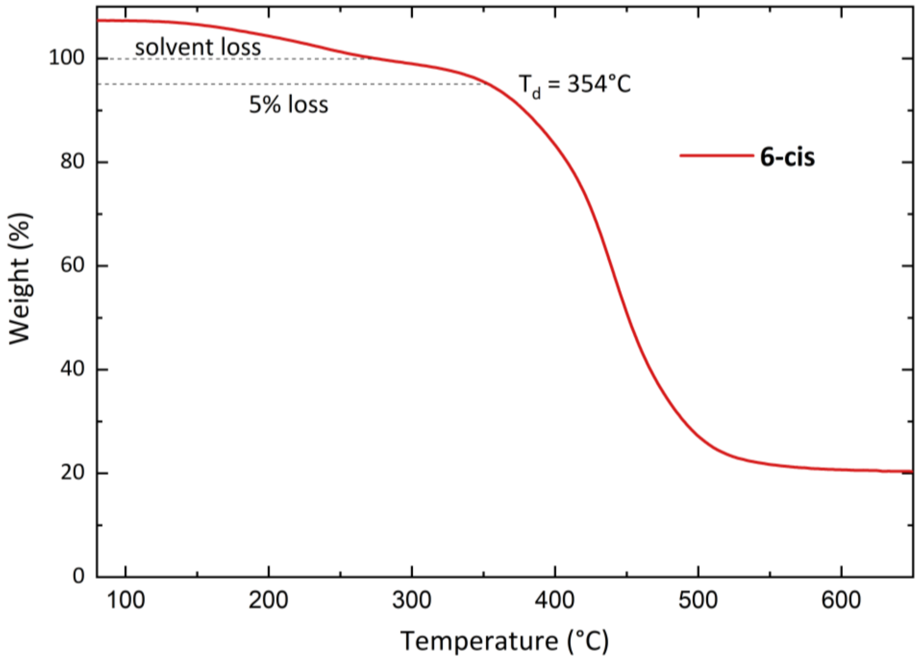


**Figure S23.** TGA of compounds **6-cis**.

# 5. Electrochemical measurements

## 5.1. Cyclic voltammetry in solution

Cyclic voltammetry (CV) measurements in solution were carried out at arbitrary concentrations of the macrocycles in 0.1 M [$n$-$Bu_4N$]$PF_6$ in DMF with a Metrohm Autolab PGSTAT101 Electrochemical Analyser interfaced to NOVA software at a scan rate of 0.1 V $s^{-1}$. A one-compartment three-electrode electrochemical cell was used for all measurements, featuring a 3 mm diameter glassy carbon working electrode, an $Ag/Ag^+$ non-aqueous quasi-reference electrode, and a Pt counter electrode. Residual oxygen was removed from the electrolyte and it was saturated with dried $N_2$ by bubbling for 20 min prior to each measurement. Ferrocene was added as an internal reference upon completion of sample measurements.

The cyclic voltammograms shown in Fig. 3a show the second cycle of the measurement. In line with best practice for solution measurements,[4] the redox potentials shown in Fig. 3a were estimated from the half-wave potential ($E^{1/2}$) when reversibility was observed and from the inflection-point potential ($E^i$) when no reversibility was observed. In addition to the estimated redox potentials, Fig. 3a also shows the cathodic and anodic peak potentials (in smaller font size).

## 5.2. Cyclic voltammetry of thin films

For thin film measurements, 5 mg $mL^{-1}$ solutions were prepared for each macrocycle. These were then dropcast onto the working electrode and the solution was allowed to evaporate to form a film. These were then measured in the same three-electrode configuration as described above for the solution state measurements, using oxygen removed and $N_2$ saturated 0.1 M [n-$Bu_4N$]$PF_6$ in $CH_3CN$ as the electrolyte.

The cyclic voltammograms in Fig. 3b show the first cycle of the measurement as the irreversible oxidation at high potential resulted in changes in the second cycle of the measurement. The redox potentials shown in Fig. 3b were estimated from the onset of the reduction.

As the coverage of the working electrode with macrocycle thin films appeared to alter the ferrocene measurements when adding ferrocene as an internal reference, a separate ferrocene measurement with cleaned electrodes was recorded as a reference for all thin-film measurements.

## 6. UV-vis absorption and photoluminescence measurements

Solution measurements were obtained using 5 µM (**6-cis** and **6-trans**) or 10 µM (PCT, **3**, **4** and **5**) solutions in chloroform, and thin film measurements were obtained from cleaned glass slides coated with 40 µL of 5 mg $mL^{-1}$ solutions using a spin coater at 1000 rpm for 1 min.

UV-vis absorption spectra were recorded on an Agilent Cary 60 UV-Vis spectrophotometer at room temperature under regular lab conditions at a data interval of 0.5 nm.

Photoluminescence (PL) spectra of the macrocycles in solution were acquired on an Agilent Cary Eclipse fluorescence spectrophotometer at a data interval of 1.0 nm, using the same solutions as for the UV-vis absorption measurements. The excitation and emission slits were set to 5 nm, and the detector voltage was set to 'high' (800 V).

**Table S1.** UV-vis absorption data.

| Molecule | $\lambda_{abs, max}$ [nm] (solution) | $\lambda_{abs, max}$ [nm] (thin film) |
|---|---|---|
| **PCT** | 307 | 318 |
| **3** | 303 | 319 |
| **4** | 308 | 315 |
| **5** | 319<br>341 (shoulder) | 325 |
| **6-cis** | 311 | 316 |
| **6-trans** | 312 | 316 |

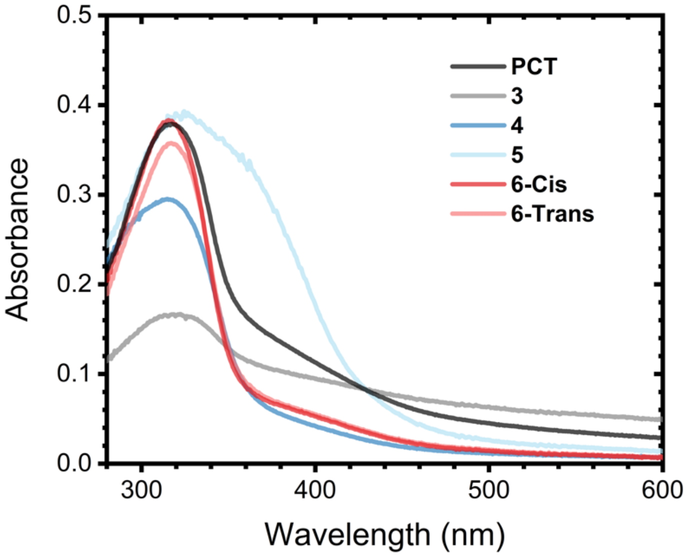


**Figure S24.** Thin-film UV-vis absorption spectra of the macrocycles.

## 7. Computational analysis

Geometry optimisations on the singlet ground state ($S_0$), the 2+ and 2- charged states, as well as the triplet ($T_1$), were carried out using the PBE0 functional with def2-SV(P) basis set and D3 dispersion correction.[5-8] For the singlet states, spin-restricted Kohn-Sham (RKS) computations were performed, and for the triplet unrestricted KS (UKS) was used. Vibrational frequency analyses at the same level were performed to verify the nature of the stationary points as minima and to obtain thermostatistical corrections for the redox potentials. Additional single-point computations were performed at the D3-PBE0/def2-SVPD level, including solvation effects using the SMD model of Truhlar and co-workers[9] to represent DMF. Redox potentials were computed from these D3-PBE0/def2-SVPD energies along with D3-PBE0/SV(P) thermostatistical corrections as described previously.[10] The excited singlet state ($S_1$) was optimised using time-dependent density functional theory (TDDFT) at the LC-BLYP/def2-SV(P) level using a range separation parameter of $\mu$ = 0.1 a.u.[11] The calculations were performed in Orca 5.0.[12]

Nucleus-independent chemical shifts (NICS)[13] were computed at the PBE0/def2-SVP level using gauge including atomic orbitals[14] as implemented in Gaussian 09.[15] NICS tensors were represented graphically using the visualisation of chemical shielding tensors (VIST) method[16] as implemented in TheoDORE 3.0[17] and using VMD as a graphical interface.[18]

Changes in the electron density for the charged states and $T_1$ were modelled by computing natural difference orbitals.[19] This analysis was performed using the "analyze_nos" functionality of TheoDORE 3.0 using the molecular orbitals of the charged and neutral states (both computed at the geometry of the charged state) as input. The analysis of $S_1$ states was done using natural transition orbitals.[20]

The underlying computational research data is available via a separate repository (DOI: 10.17028/rd.lboro.22306207): Orca input/output files for geometry optimisations, frequency analyses, and solvated single-point computations; Gaussian input/output files for NICS computations.

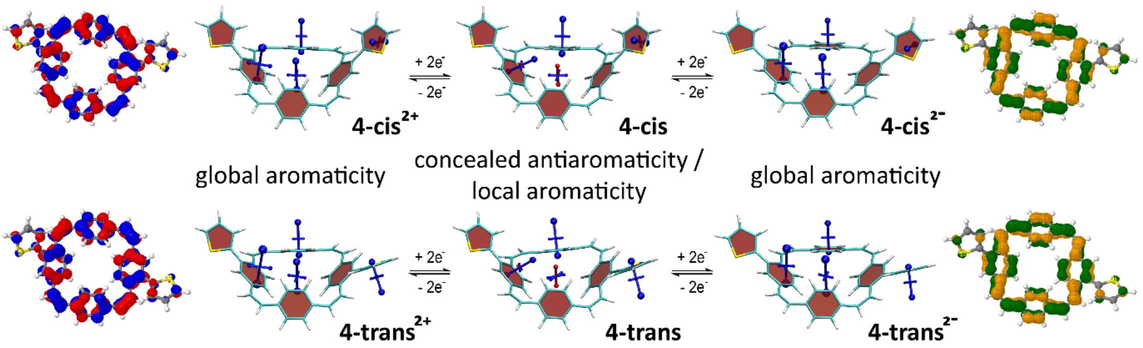


**Figure S25.** Analysis of the neutral and doubly charged states for **4-cis** and **4-trans**. Centre: VIST plots. Left and right: Dominant natural difference orbitals (NDOs) between the neutral states and the dications (blue/red NDOs for electron detachment) and the dianions (green/orange NDOs for attachment).

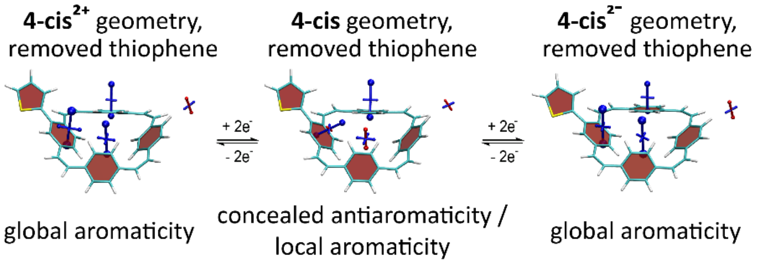


**Figure S26.** VIST plots for the neutral and doubly charged states of a macrocycle at the geometry of **4-cis** but with removed thienyl unit. The increased deshielding in the doubly charged states shown by the tensor of the removed thienyl unit (at the outside of the macrocyclic conjugated system) is a result of the macrocyclic diatropic currents in these states. This deshielding effect can explain the reduced shielding in this position if the thienyl unit is present, an effect also observed for the other aryl-substituted macrocycles.

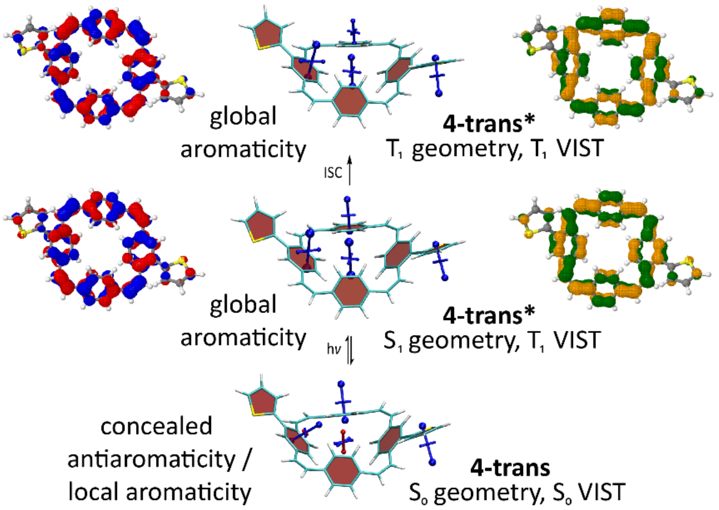


**Figure S27.** Analysis of the $S_0$, $S_1$, and $T_1$ states for **4-trans**. Centre: VIST plots (using $T_1$ also at the $S_1$ geometry). Left and right: Dominant natural transition/difference orbitals (NTOs/NDOs) for the $S_1$ and $T_1$ states (blue/red for electron detachment; green/orange for attachment).

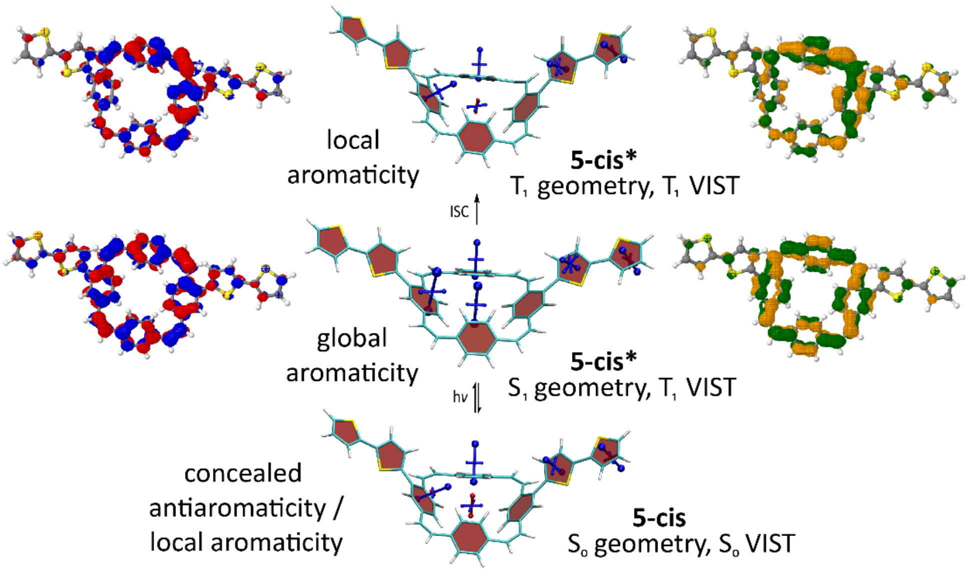


**Figure S28.** Analysis of the $S_0$, $S_1$, and $T_1$ states for **5-cis**. Centre: VIST plots (using $T_1$ also at the $S_1$ geometry). Left and right: Dominant natural transition/difference orbitals (NTOs/NDOs) for the $S_1$ and $T_1$ states (blue/red for electron detachment; green/orange for attachment).

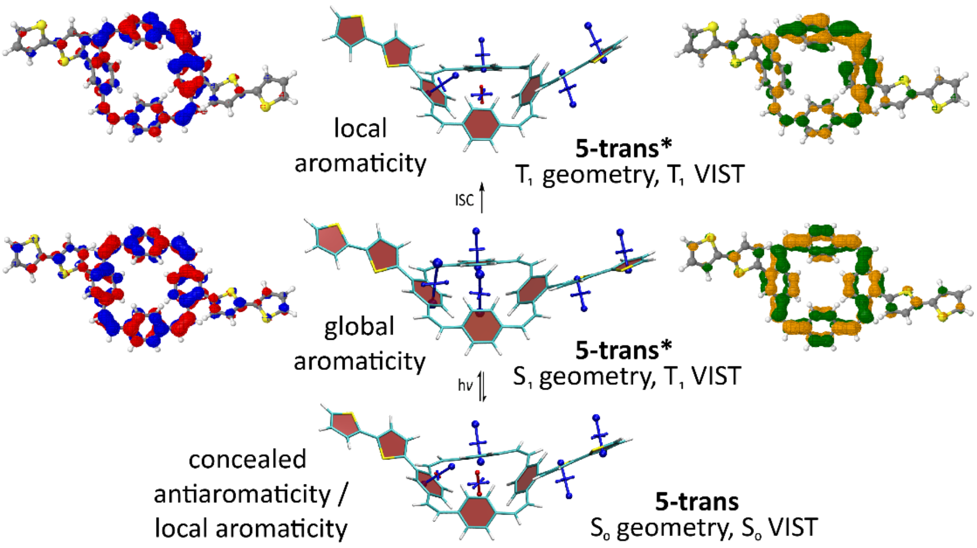


**Figure S29.** Analysis of the $S_0$, $S_1$, and $T_1$ states for **5-trans**. Centre: VIST plots (using $T_1$ also at the $S_1$ geometry). Left and right: Dominant natural transition/difference orbitals (NTOs/NDOs) for the $S_1$ and $T_1$ states (blue/red for electron detachment; green/orange for attachment).

**Table S2.** NICS values (in ppm) corresponding to the tensor component perpendicular to the plane of the PCT core at the centre of the molecule (Centre) as well as to the main component of the tensors above the substituted (Ph1) and unsubstituted phenylene subunits (Ph2) of the PCT core, and above the thienyl/thienylene unit directly linked to the PCT core (Thio) and further away (Thio2).

| Molecule | State | Centre | Ph1 | Ph2 | Thio | Thio2 |
|---|---|---|---|---|---|---|
| **4-cis** | 0 | 11.1 | -23.3 | -19.8 | -25.4 | |
| | 2+ | -24.2 | -19.7 | -34.6 | -21.8 | |
| | 2- | -34.2 | -17.2 | -36.5 | -19.4 | |
| | triplet | 4.4 | -14.1 | -20.2 | -25.5 | |
| | singlet | -33.5 | -19.2 | -36.8 | -22.0 | |
| **4-trans** | 0 | 10.9 | -23.9 | -20.0 | -25.1 | |
| | 2+ | -26.6 | -21.5 | -37.1 | -21.3 | |
| | 2- | -34.7 | -18.5 | -36.8 | -19.7 | |
| | triplet | -22.4 | -10.7 | -30.3 | -24.1 | |
| | singlet | -33.7 | -20.6 | -37.0 | -21.8 | |
| **5-cis** | 0 | 11.1 | -23.2 | -19.6 | -19.4 | -22.9 |
| | 2+ | -9.0 | -19.5 | -23.8 | -14.2 | -18.9 |
| | 2- | -20.6 | -16.4 | -27.0 | -11.8 | -18.4 |
| | triplet | 4.7 | -13.9 | -20.0 | -19.1 | -22.9 |
| | singlet | -32.1 | -18.2 | -35.8 | -14.6 | -20.8 |
| **5-trans** | 0 | 11.0 | -23.7 | -19.8 | -18.9 | -23.0 |
| | 2+ | -10.8 | -21.0 | -25.6 | -14.1 | -19.2 |
| | 2- | -22.0 | -18.7 | -28.5 | -11.1 | -18.8 |
| | triplet | 6.2 | -14.7 | -19.9 | -19.3 | -22.7 |
| | singlet | -32.9 | -20.5 | -36.5 | -16.1 | -21.5 |
| **4-cis** with removed thienyl unit | 0 | 10.9 | -23.5 | -19.8 | 2.9 | |
| | 2+ | -27.4 | -20.6 | -37.4 | 6.7 | |
| | 2- | -35.7 | -17.2 | -37.3 | 7.7 | |

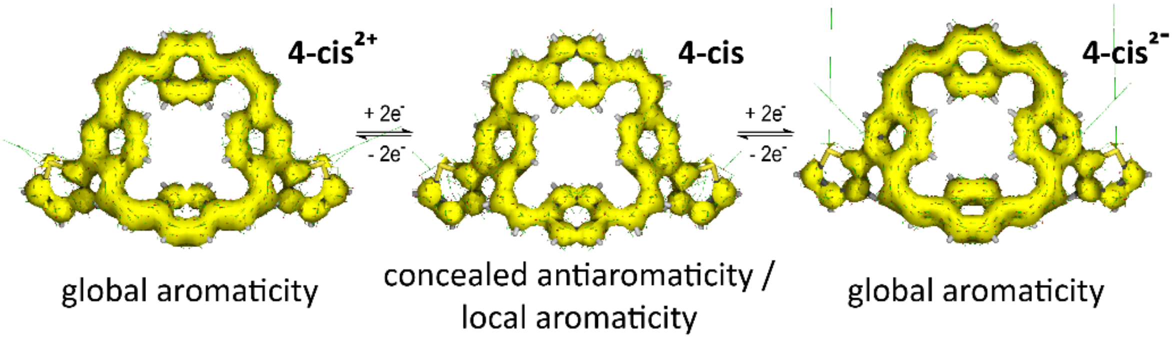


**Figure S30.** ACID plots for **4-cis** in the neutral and doubly charged states.

**Table S3.** Computed redox potentials for the reduction (0 to -2) and oxidation (0 to +2) of thienyl- and bithienyl-substituted PCTs vs. $Fc/Fc^+$ using a voltage of 4.7 V for the reference electrode.

| | ***-2/0*** | ***0/+2*** |
|---|---|---|
| **4-cis** | -2.077 | 0.596 |
| **4-trans** | -2.080 | 0.607 |
| **5-cis** | -2.071 | 0.598 |
| **5-trans** | -2.083 | 0.633 |

## 8. References


1 P. Liu, Y. Chen, J. Deng and Y. Tu, *Synthesis*, 2001, **2001**, 2078-2080. DOI:10.1055/s-2001-18060

2 T. J. Katz, A. Sudhakar, M. F. Teasley, A. M. Gilbert, W. E. Geiger, M. P. Robben, M. Wuensch and M. D. Ward, *J. Am. Chem. Soc.*, 1993, **115**, 3182-3198. DOI:10.1021/ja00061a018

3 B. Thulin, O. Wennerström and H.-E. Högberg, *Acta Chem. Scand., Ser. B*, 1975, **29**, 138-139. DOI:10.3891/acta.chem.scand.29b-0138

4 E. M. Espinoza, J. A. Clark, J. Soliman, J. B. Derr, M. Morales and V. I. Vullev, *J. Electrochem. Soc.*, 2019, **166**, H3175-H3187. DOI:10.1149/2.0241905jes

5 J. P. Perdew, K. Burke and M. Ernzerhof, *Phys. Rev. Lett.*, 1996, **77**, 3865-3868. DOI:10.1103/PhysRevLett.77.3865

6 C. Adamo and V. Barone, *J. Chem. Phys.*, 1999, **110**, 6158-6170. DOI:10.1063/1.478522

7 F. Weigend and R. Ahlrichs, *Phys. Chem. Chem. Phys.*, 2005, **7**, 3297-3305. DOI:10.1039/B508541A

8 S. Grimme, J. Antony, S. Ehrlich and H. Krieg, *J. Chem. Phys.*, 2010, **132**, 154104. DOI:10.1063/1.3382344

9 A. V. Marenich, C. J. Cramer and D. G. Truhlar, *J. Phys. Chem. B*, 2009, **113**, 6378-6396. DOI:10.1021/jp810292n

10 S. Eder, B. Ding, D. B. Thornton, D. Sammut, A. J. P. White, F. Plasser, I. E. L. Stephens, M. Heeney, S. Mezzavilla and F. Glöcklhofer, *Angew. Chem., Int. Ed.*, 2022, **61**, e202212623. DOI:10.1002/anie.202212623

11 Y. Tawada, T. Tsuneda, S. Yanagisawa, T. Yanai and K. Hirao, *J. Chem. Phys.*, 2004, **120**, 8425-8433. DOI:10.1063/1.1688752

12 F. Neese, *WIREs Computational Molecular Science*, 2022, **12**, e1606. DOI:10.1002/wcms.1606

13 P. v. R. Schleyer, C. Maerker, A. Dransfeld, H. Jiao and N. J. R. van Eikema Hommes, *J. Am. Chem. Soc.*, 1996, **118**, 6317-6318. DOI:10.1021/ja960582d

14 J. R. Cheeseman, G. W. Trucks, T. A. Keith and M. J. Frisch, *J. Chem. Phys.*, 1996, **104**, 5497-5509. DOI:10.1063/1.471789

15 M. J. Frisch, G. W. Trucks, H. B. Schlegel, G. E. Scuseria, M. A. Robb, J. R. Cheeseman, G. Scalmani, V. Barone, B. Mennucci, G. A. Petersson, H. Nakatsuji, M. Caricato, X. Li, H. P. Hratchian, A. F. Izmaylov, J. Bloino, G. Zheng, J. L. Sonnenberg, M. Hada, M. Ehara, K. Toyota, R. Fukuda, J. Hasegawa, M. Ishida, T. Nakajima, Y. Honda, O. Kitao, H. Nakai, T. Vreven, J. J. A. Montgomery, J. E. Peralta, F. Ogliaro, M. Bearpark, J. J. Heyd, E. Brothers, K. N. Kudin, V. N. Staroverov, T. Keith, R. Kobayashi, J. Normand, K. Raghavachari, A. Rendell, J. C. Burant, S. S. Iyengar, J. Tomasi, M. Cossi, N. Rega, J. M. Millam, M. Klene, J. E. Knox, J. B. Cross, V. Bakken, C. Adamo, J. Jaramillo, R. Gomperts, R. E. Stratmann, O. Yazyev, A. J. Austin, R. Cammi, C. Pomelli, J. W. Ochterski, R. L. Martin, K. Morokuma, V. G. Zakrzewski, G. A. Voth, P. Salvador, J. J. Dannenberg, S. Dapprich, A. D. Daniels, O. Farkas, J. B. Foresman, J. V. Ortiz, J. Cioslowski and D. J. Fox, *Gaussian 09, Revision E.01*, Wallingford, CT, 2013.

16 F. Plasser and F. Glöcklhofer, *Eur. J. Org. Chem.*, 2021, **2021**, 2529-2539. DOI:10.1002/ejoc.202100352

17 F. Plasser, *J. Chem. Phys.*, 2020, **152**, 084108. DOI:10.1063/1.5143076

18 W. Humphrey, A. Dalke and K. Schulten, *J. Mol. Graph.*, 1996, **14**, 33-38. DOI:10.1016/0263-7855(96)00018-5

19 F. Plasser, M. Wormit and A. Dreuw, *J. Chem. Phys.*, 2014, **141**, 024106. DOI:10.1063/1.4885819

20 R. L. Martin, *J. Chem. Phys.*, 2003, **118**, 4775-4777. DOI:10.1063/1.1558471
